# SOX1 acts as a tumor hypnotist rendering nasopharyngeal carcinoma cells refractory to chemotherapy

**DOI:** 10.1038/s41420-023-01479-x

**Published:** 2023-06-27

**Authors:** Xin-Xing Lei, Shu-Lan Wang, Ying Xia, Min Yan, Bin He, Bo Wang, Zi-Jie Long, Quentin Liu

**Affiliations:** 1 State Key Laboratory of Oncology in South China, Collaborative Innovation Center for Cancer Medicine, Guangdong Key Laboratory of Nasopharyngeal Carcinoma Diagnosis and Therapy, Sun Yat-sen University Cancer Center, 510060 Guangzhou, P. R. China; 2 Department of Oncology, The Seventh Affiliated Hospital, Sun Yat-sen University, 518107 Shenzhen, P. R. China; 3 State Key Laboratory of Organ Failure Research, Guangdong Provincial Key Laboratory of Viral Hepatitis Research, Department of Infectious Diseases, Nanfang Hospital, Southern Medical University, 510515 Guangzhou, P. R. China; 4 Department of Hematology, The Third Affiliated Hospital, Sun Yat-sen University, 510630 Guangzhou, P. R. China

**Keywords:** Tumour biomarkers, Prognostic markers, Cancer therapeutic resistance, Cancer models

## Abstract

SOX1, a well-known tumor suppressor, delays malignant progression in most cancer types. However, high expression of SOX1 in late-stage head and neck squamous cell carcinoma leads to poor prognosis. In this study, we show that SOX1 induces nasopharyngeal carcinoma (NPC) cells to enter a quiescent state. Using a model that mimics therapeutic resistance and tumor recurrence, a subpopulation of SOX1-induced NPC cells is refractory to paclitaxel, a cell cycle-specific chemotherapy drug. These cells maintain a quiescent state with decreased translational activity and down-regulated cell growth potential. However, once SOX1 expression is decreased, the NPC cells recover and enter a proliferative state. The chemotherapy resistance induced by SOX1 can not pass to next generation, as the cells that undergo re-proliferation become sensitive to paclitaxel again. Moreover, SOX1 directly binds to the promoter region of the *MYC* gene, leading to transcriptional suppression. When switching to a paclitaxel-free culture environment, the cells with decreased levels of SOX1 re-express MYC, resulting in increased abundance of proliferative cancer cells. Our study presents an evolutionary trade-off between tumor growth and chemoresistance orchestrated by SOX1-MYC in NPC. Basing on the dynamic role of SOX1 in different stages of cancer development, SOX1 would be regarded as a “tumor hypnotist”.

## Introduction

Nasopharyngeal carcinoma (NPC) is a prevalent disease in Southern China and Southeast Asian countries, with concurrent chemoradiotherapy being a key treatment option. However, chemoresistance poses a significant challenge to the successful therapy in NPC, as 10–20% of patients experience recurrence after initial treatment [1], with 70–80% of recurrent NPC cases being locally advanced [2]. Thus, gaining a comprehensive understanding of the mechanisms of NPC chemoresistance is crucial.

Current study indicates that quiescent cancer cells (QCCs) may be responsible for NPC recurrence [3], as QCCs are dormant for long periods of time and account for resistance to conventional cancer treatments [4]. Moreover, QCCs can re-enter the cell cycle and proliferate, leading to cancer progression and metastasis [5]. To tackle this challenging problem, numerous studies have focused on the mechanisms that regulate initiation and termination of cell quiescence [6]. Various intracellular markers, such as Ki-67, c-Myc, Cyclin D, p27Kip1, or Rb, have been applied to detect the quiescent state of cancer cells, while most in vitro models of QCCs rely on alteration of tumor micro-environment, such as nutrient deprivation, hypoxia, contact inhibition, or anti-cancer treatment [7]. However, the intrinsic factors regulating QCCs, independent of extracellular environmental cues, remain unknown.

SOX1, a transcription factor that primarily functions in neurogenesis, has been shown to act as a tumor suppressor in several cancer types, including NPC [8]. In the current study, we present a new model that mimics QCC by overexpression of SOX1 in NPC cells. The term “tumor suppressor” suggests that the genes have inhibitory effects on cancer cells proliferation, nevertheless, some of these genes in turn favors cancer cells survival under therapeutic interventions. Therefore, these genes may be re-classified as “tumor hypnotists” attributing to their dynamic roles in different tumor micro-environments.

## Results

### Tumor suppressor SOX1 contributes to poor prognosis in several cancer types

To gain a better understanding of the role of SOX1 in cancer cells, we had reviewed the existing studies on SOX1 in human cancer. Overexpression of SOX1 could inhibit cell proliferation, invasion, and tumor formation in most cancer types, such as NPC [8], hepatocellular carcinoma [9], cervical cancer [10], lung cancer [11], gastric cancer [12], breast cancer [13] and cholangiocarcinoma [14] (Table 1). Conversely, SOX1 acted as an oncogene in glioblastoma [15]. The analysis of the TCGA database showed that *SOX1* expression was low in most cancer types, except for glioblastoma tissues (Supplementary Fig. S1A). Moreover, <5% of tumor samples in the TCGA cohorts had mutations in the *SOX1* gene (Supplementary Fig. S1B), indicating that SOX1 might exert its function through alteration of its own RNA/protein levels.

**Table 1:** SOX1-associated research achievements on different cancer types up to date.

Authors	Year	Tumor types	SOX1 expression in tumor	Functions of SOX1			
				Proliferation	Invasion	Tumor formation	Apoptosis
Tsao, C.-M. et al.	2012	Hepatocellular carcinoma	Low	Inhibition	Inhibition	Inhibition	
Lin, Y. W. et al.	2013	Cervical cancer	Low	Inhibition	Inhibition	Inhibition	
Guan, Z. et al.	2014	Nasopharyngeal carcinoma	Low	Inhibition	Inhibition	Inhibition	
Li, N. & Li, S	2015	Lung cancer	Low	n.s.	Inhibition	n.s.	
Chen, J. et al.	2016	Gastric cancer	Low	Inhibition			Promotion
Song, L. et al.	2016	Breast cancer	Low	Inhibition	Inhibition		
Garcia, I. et al.	2017	Glioblastoma	High	Promotion		Promotion	
Chen, Y. et al.	2021	Cholangiocarcinoma	Low	Inhibition		Inhibition	n.s.

*n.s.*: not significant.

Unexpectedly, patients with high levels of *SOX1* expression in several cancer tissues had a poorer prognosis, as compared to those with low levels of *SOX1* (Supplementary Fig. S2A, B). On the other hand, patients with high levels of *SOX1* in lower grade glioma had better outcomes (Supplementary Fig. S2A, B). Moreover, we noted that patients who experienced “new neoplasm event post initial therapy” in head and neck squamous cell carcinoma (HNSC), colorectal cancer, and tenosynovial giant cell tumor had elevated *SOX1* expression in their original tumors (Supplementary Fig. S2C, Supplementary Table S1). In addition, high levels of *SOX1* were related to poor prognosis in late-stage HNSC patients, whereas no significant effect was observed in early-stage patients (Supplementary Fig. S2D, E). Our findings raised the intriguing question why a tumor suppressor like SOX1 could have a negative regulation on the prognosis of late-stage HNSC patients.

### SOX1 reduces translational activity and DNA replication of NPC cells

**Fig. 1:**
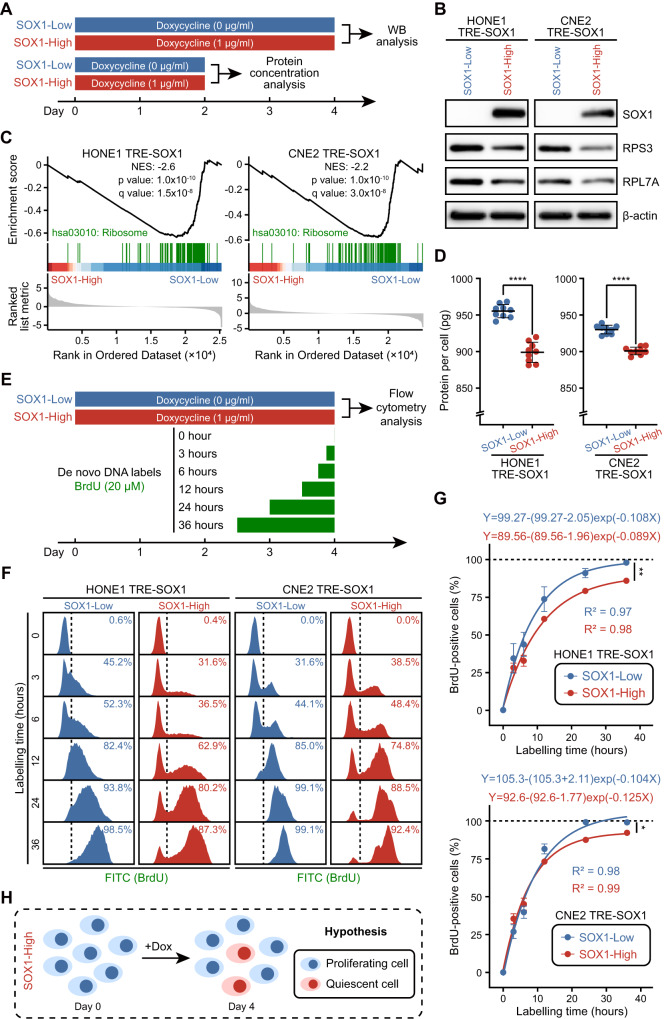
SOX1 decreases ribosome pathway and DNA replication in NPC cells. **A** An experimental timeline for doxycycline (Blue block: 0 µg/ml, red block: 1 µg/ml) schedules used to control the expression of SOX1 in cells (HONE1 TRE-SOX1 or CNE2 TRE-SOX1). On day 2 and day 4, the cells were counted and proteins were extracted for protein concentration and western blot analysis, respectively. **B** Western blot analysis of SOX1, RPS3, RPL7A, and β-actin expression in cells. β-actin was used as a control. **C** Gene set enrichment analysis (GSEA) of ranked genes from cells. Gene set “hsa03010: Ribosome” of KEGG pathways was presented. NES normalized enrichment score. **D** Scatter dot plots showing the average protein content per cell. **E** An experimental timeline for doxycycline (Blue block: 0 µg/ml, red block: 1 µg/ml) schedules used to control the expression of SOX1 in cells (HONE1 TRE-SOX1 or CNE2 TRE-SOX1). Both groups of cells were treated with 20 µM BrdU for varying durations, and on day 4, BrdU-positive cells were determined by flow cytometry analysis. **F** Histograms showing the percentage of BrdU-negative or -positive cells in “SOX1-High” and “SOX1-Low” groups of cells. **G** Time courses of the percentage of BrdU-positive cells in BrdU incorporation assays, which were fitted by an exponential plateau equation. **H** An illustration of model hypothesis that overexpression of SOX1 induces quiescent NPC cells. Dox Doxycycline.

To find out how tumor suppressor SOX1 promoted cancer cell survival after therapy, we conducted RNA-seq analysis on NPC cells with high versus low expression of SOX1 (Supplementary Fig. S3A). Our previous work [16] had established stable clones (HONE1 TRE-SOX1 and CNE2 TRE-SOX1) in which SOX1 overexpression could be induced by doxycycline treatment under the Tet-ON system. The gene expression profiles of the two groups were well-clustered based on the results of principal component analysis (PCA) analysis (Supplementary Fig. S3B). The induction of SOX1 expression by doxycycline was observed in the “SOX1-High” group (Fig. 1A, B, Supplementary Fig. S3C). Gene set enrichment analysis (GSEA) showed that the ribosome pathway was significantly enriched in “SOX1-Low” group cells (Fig. 1C, Supplementary Fig. S3D, Supplementary Tables S2, S3). We observed decreased protein levels of RPS3 and RPL7A in “SOX1-High” group cells, which are part of the small and large ribosomal subunits, respectively (Fig. 1B). Reduced protein contents were also observed in “SOX1-High” group cells (Fig. 1A, D), indicating that SOX1 decreased translational activity in NPC cells. In addition, we incorporated 5-Bromo-2′-deoxyuridine (BrdU) to mark the newly synthesized DNA in cells (Fig. 1E). Nearly all cells in the “SOX1-Low” group were marked at 36 h, indicating that the cells had passed through the G1/S transition during that time (Fig. 1F, G). However, a minority of cells (8–15%) in the “SOX1-High” group remained unmarked for BrdU, suggesting that these cells were still in the G0/G1 phase (Fig. 1F, G).

Based on the evidence presented above, we proposed a hypothesis to explain the conflicting role of tumor suppressor SOX1 in promoting survival of cancer cells after therapy. To understand the functions of SOX1, previous studies mostly relied on cell biology assays or mouse tumor transplantation experiments conducted in a stress-free environment (Table 1). Similarly, tumors in patients grow and spread until they are treated with chemotherapy or other therapeutic interventions. Our hypothesis proposes that NPC cells with low expression of SOX1 remain in a highly proliferative state, while overexpression of SOX1 may play a role in inducing quiescence in NPC cells, leading to drug resistance. Therefore, SOX1 may function as a “tumor hypnotist” (Fig. 1H), facilitating cancer cell survival upon exposure to chemotherapy and other unfavorable conditions.

### SOX1-induced QCCs are refractory to cell cycle-specific chemotherapy

**Fig. 2:**
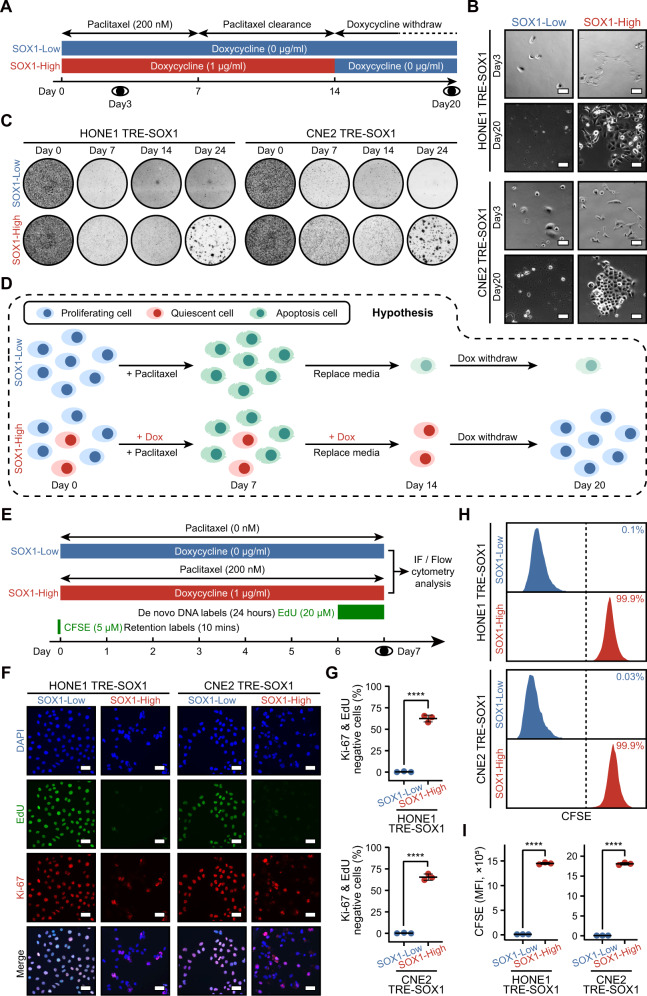
A model of SOX1-induced quiescent NPC cells under cell cycle-specific chemotherapy. **A** An experimental timeline for multiple schedules of paclitaxel/doxycycline treatment in cells (HONE1 TRE-SOX1 or CNE2 TRE-SOX1). The cells were treated with doxycycline (Blue block: 0 µg/ml, red block: 1 µg/ml) to control SOX1 expression. The “SOX1-High” group cells were pre-treated with doxycycline for 4 days and terminated on day 14, while both groups of cells were treated with 200 nM paclitaxel for 7 days and cultured in a paclitaxel-free environment. **B** Bright field images of the cells on day 3 and day 20. Scale bar = 50 µm. **C** Colony formation assay of the cells on day 0, 7, 14, and 24. **D** An illustration of model hypothesis mimicking SOX1-induced QCCs. The “SOX1-Low” group cells were used as negative control, while “SOX1-High” group cells underwent killing of proliferative cells (day 0–7), clearance of intracellular paclitaxel (day 7–14), and reactivation of QCCs (day 14–20). Dox Doxycycline. **E** An experimental timeline for the EdU/CFSE/paclitaxel/doxycycline multiple schedules. Cells (HONE1 TRE-SOX1 or CNE2 TRE-SOX1) were treated with doxycycline (Blue block: 0 µg/ml, red block: 1 µg/ml) to control SOX1 expression. The “SOX1-Low” group cells were cultured in a paclitaxel-free environment and used as positive (EdU labeling) or negative control (CFSE labeling). The “SOX1-High” group cells were pre-treated with doxycycline for 4 days and terminated on day 7, and treated with 200 nM paclitaxel for 7 days. Both groups of cells were treated with 20 µM EdU on the last day or labeled by 5 µM CFSE for 10 min before paclitaxel treatment. For EdU labeling, cells were fixed and stained with Azide-fluor 488, Ki-67 antibody and DAPI. For CFSE labeling, cells were collected for flow cytometry analysis. **F** Representative images of EdU incorporation assays and Ki-67 staining in “SOX1-High” or “SOX1-Low” group cells. Blue: DAPI, Green: EdU, Red: Ki-67. Scale bar = 50 µm. **G** Scatter dot plots showing the percentage of EdU&Ki-67 double negative cells in total. **H** Histograms showing fluorescence intensity of CFSE in “SOX1-High” or “SOX1-Low” group cells. The numbers indicate the percentage of cells that retained CFSE labeling in the total cell population. **I** Scatter dot plots showing mean fluorescence intensity (MFI) of CFSE in cells.

To test our hypothesis, NPC cells were cultured in a stressful environment where chemotherapeutics was applied. Two NPC cell lines (HONE1 and CNE2) were subjected to gradient concentrations of paclitaxel, a cell cycle-specific chemotherapy (chemo) drug, and even the lowest concentration (20 nM) resulted in cellular morphological changes by day 3. These alterations were histologically characterized as floating (apoptosis), spherical (G2/M arrest), and giant (cytokinesis failure) phenotypes (Supplementary Fig. S4). A pharmacokinetic study showed that stable concentration ranges of paclitaxel from 10 to 200 ng/ml are found in human plasma [17], thus cells were treated with 200 nM (171 ng/ml) paclitaxel for 7 days to completely eradicate all proliferative NPC cells (Fig. 2A). Under the intervention of paclitaxel, we observed morphologic differences between cells with high versus low expression of SOX1. On day 3, the “SOX1-Low” group showed similar phenotypes with results in Supplementary Fig. S4, while the “SOX1-High” group resulted in slender and fusiform phenotypes (Fig. 2B). To clear up the retained paclitaxel within cells, the medium was renewed every 2 days after withdrawal of paclitaxel. Doxycycline was not supplied anymore in the “SOX1-High” group on day 14 (Fig. 2A). Clonal clustered cells were present after 6 days withdrawal of doxycycline in the “SOX1-High” group, while no cells were reactivated in the “SOX1-Low” group (Fig. 2B). Colony formation assay also showed distinct clonal clusters in the “SOX1-High” group on day 24 (Fig. 2C). As a result, the NPC cells could potentially switch from a proliferative to a quiescent state upon the expression of SOX1, while the QCCs might regain their ability to proliferate as the expression of SOX1 decreased (Fig. 2D). In the “SOX1-Low” group, proliferative cells underwent cell-cycle phase under paclitaxel treatment and lived no longer than 7 days, mimicking tumor regression. However, in the “SOX1-High” group, the QCCs were able to undergo stress and reactivate, mimicking tumor recurrence.

Furthermore, we utilized cisplatin, a cell-cycle nonspecific chemo drug, in the same schedule of the model (Supplementary Fig. S5A). On day 3, we observed morphological changes, with no difference between cells with high versus low expression of SOX1 (Supplementary Fig. S5B). Unlike paclitaxel, cisplatin could kill cells during any phase of the cell cycle, including the QCCs induced by SOX1, and no recovering cells could form clonal clusters during the reactivating phase (Supplementary Fig. S5B). The fate of the “SOX1-High” group was the same as that of the “SOX1-Low” group, which strengthened the evidence that overexpression of SOX1 promoted cells to enter a quiescent state under cell cycle-specific therapy.

To confirm the quiescent status of cells in our model, we labeled de novo DNA in cells using 5-ethynyl-2′-deoxyuridine (EdU) (Fig. 2E), combined with a proliferation marker for cancer cells (Ki-67). In the “SOX1-Low” group, nearly all nuclei were double positive for EdU and Ki-67, while in the “SOX1-High” group, over 60% were double negative (Fig. 2F, G). We also labeled cells with carboxyfluorescein diacetate succinimidyl ester (CFSE) (Fig. 2E) and observed a high intensity of CFSE signal in “SOX1-High” group cells after 7 days (Fig. 2H, I). Taken together, these results provide further evidence of the quiescent cells existed in our model.

### Dynamic expression of SOX1 alters the fate of QCCs

**Fig. 3:**
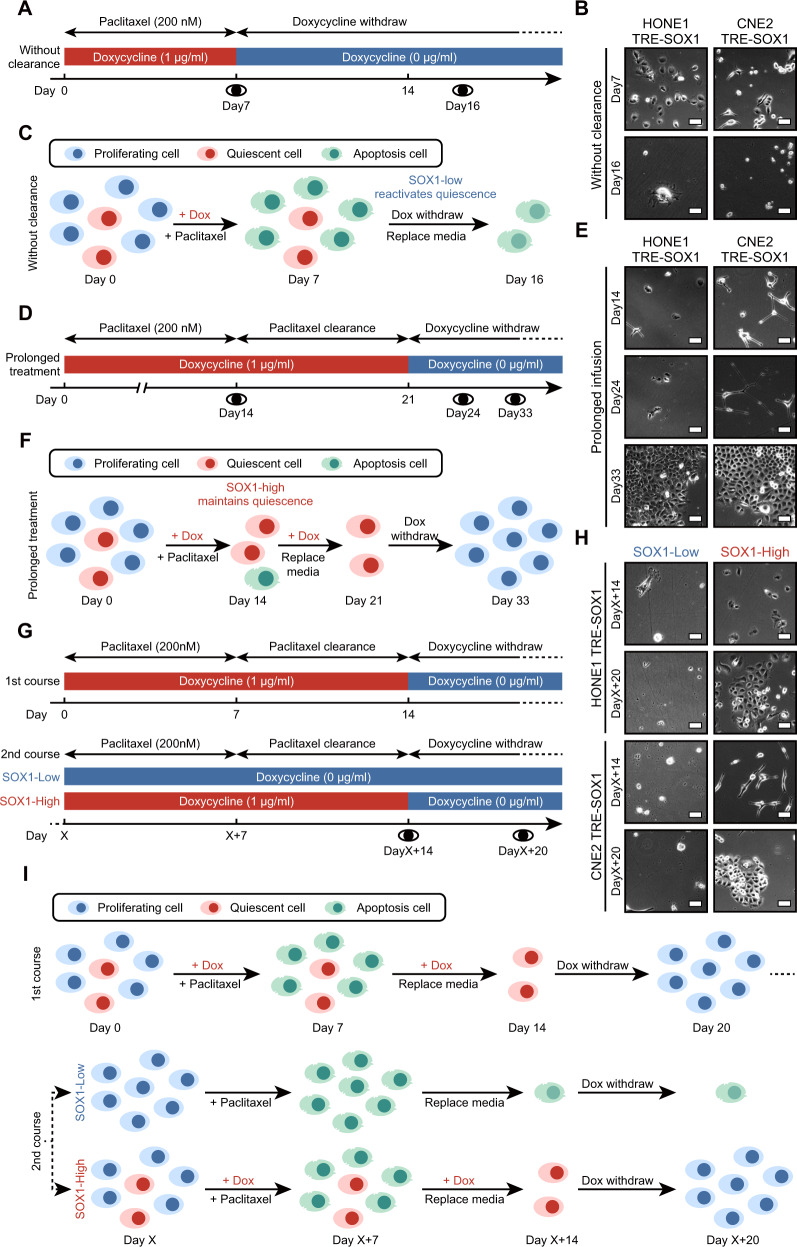
Paclitaxel resistance induced by SOX1 is persistent and reversible. **A** An experimental timeline for multiple schedules of paclitaxel/doxycycline treatment in cells (HONE1 TRE-SOX1 or CNE2 TRE-SOX1). The cells were treated with doxycycline (Blue block: 0 µg/ml, red block: 1 µg/ml) to control SOX1 expression. The cells were pre-treated with doxycycline for 4 days and terminated on day 7. During this time, the cells were treated with 200 nM paclitaxel for 7 days followed by culturing in a paclitaxel-free environment. **B** Bright field images of the cells on day 7 and day 16 after treatment. Scale bar = 50 µm. **C** An illustration of the model mimicking SOX1-induced QCCs. The “without clearance” group cells underwent killing of proliferative cells (day 0–7), and reactivation of QCCs (day 7–16). Dox Doxycycline. **D** An experimental timeline for multiple schedules of paclitaxel/doxycycline treatment in cells (HONE1 TRE-SOX1 or CNE2 TRE-SOX1). The cells were treated with doxycycline (Blue block: 0 µg/ml, red block: 1 µg/ml) to control SOX1 expression. The cells were pre-treated with doxycycline for 4 days and terminated on day 21. During this time, the cells were treated with 200 nM paclitaxel for 14 days followed by culturing in a paclitaxel-free environment. **E** Bright field images of the cells on day 14, day 24, and day 33 after treatment. Scale bar = 50 µm. **F** An illustration of the model mimicking SOX1-induced QCCs for the “prolonged treatment” group cells. The cells underwent killing of proliferative cells (day 0–14), clearance of intracellular paclitaxel (day 14–21), and reactivation of QCCs (day 21–33). Dox Doxycycline. **G** An experimental timeline for multiple schedules of paclitaxel/doxycycline treatment in cells (HONE1 TRE-SOX1 or CNE2 TRE-SOX1). The cells were treated with doxycycline (Blue block: 0 µg/ml, red block: 1 µg/ml) to control SOX1 expression. For the first course of paclitaxel, the cells were pre-treated with doxycycline for 4 days and terminated on day 14. Meanwhile, cells were treated with 200 nM paclitaxel for 7 days and cultured in a paclitaxel-free environment. After reactivating quiescent cells for more than 10 days, the second course of paclitaxel was administered on day “X”. The “SOX1-High” group cells were pre-treated with doxycycline for 4 days and terminated on day 14. Meanwhile, both groups of cells were treated with 200 nM paclitaxel for 7 days followed by culturing in a paclitaxel-free environment. **H** Bright field images of the cells on day “X + 14” and day “X + 20” after treatment. Scale bar = 50 µm. **I** An illustration of the model mimicking SOX1-induced QCCs. NPC cells underwent killing of proliferative cells (day 0–7), clearance of intracellular paclitaxel (day 7–14), and reactivation of QCCs (day 14–“X”) for the first course of paclitaxel. For the second course, the “SOX1-Low” group cells were used as negative control, while the “SOX1-High” group cells underwent killing of proliferative cells (day “X”−“X + 7”), clearance of intracellular paclitaxel (day “X + 7”−“X + 14”), and reactivation of QCCs (day “X + 14”−“X + 20”). Dox Doxycycline.

We eliminated the “clearance of intracellular paclitaxel” stage to immediately awaken cells after paclitaxel treatment, as shown in the schedule (Fig. 3A). However, during the “reactivation of QCCs” stage on day 16, we did not observe any clonal cell clusters (Fig. 3B), as the QCCs were reactivated too early before the paclitaxel within the cells was cleared up (Fig. 3C).

Even after multiple courses of chemotherapy or radiotherapy, the QCCs persist in the body. To address this issue, we extended the “killing of proliferative cells” stage from 7 to 14 days (Fig. 3D). After eliminating all proliferative cells, we observed the emergence of clonal cell clusters (Fig. 3E), indicating that persistent high expression of SOX1 maintained in QCCs facilitated their ability to survive long-term paclitaxel treatment (Fig. 3F). Although prolonged exposure to the drug or multiple treatment cycles may have potential benefits for patients, our model highlights the challenges in the treatment of cancer, as it reduces the effectiveness of paclitaxel for the long-term persistent QCCs.

Many patients experience tumor recurrence due to drug resistance, as cells in tumors evolve, and adapt to a stressful environment. In such cases, cancer cells that acquire drug resistance can grow again unless another therapeutic schedule is implemented. To investigate whether reactivated NPC cells in our model had acquired resistance to paclitaxel, we designed the following experimental schedule: NPC cells were reactivated as before, followed by a second induction of SOX1 to test for drug resistance (Fig. 3G). As before, all cells in the “SOX1-Low” group were died, while QCCs in the “SOX1-High” group survived from paclitaxel treatment on day “X + 14” (Fig. 3H). We reduced the SOX1 level in these QCCs and observed the emergence of clonal cell clusters again on day “X + 20” (Fig. 3H). The cells which survived from the initial paclitaxel treatment did not acquire the ability to resist paclitaxel. Instead, overexpression of SOX1 enabled these cells to revert to a quiescent state and evade the subsequent attack of paclitaxel (Fig. 3I). The mechanisms of drug resistance in tumor cells are complex and multifactorial, but mainly attributed to genetic alterations. However, drug resistance mediated by SOX1 is reversible, suggesting non-genetic mechanism for disease recurrence.

### Decreased expression of SOX1 determines the reactivation of QCCs

**Fig. 4:**
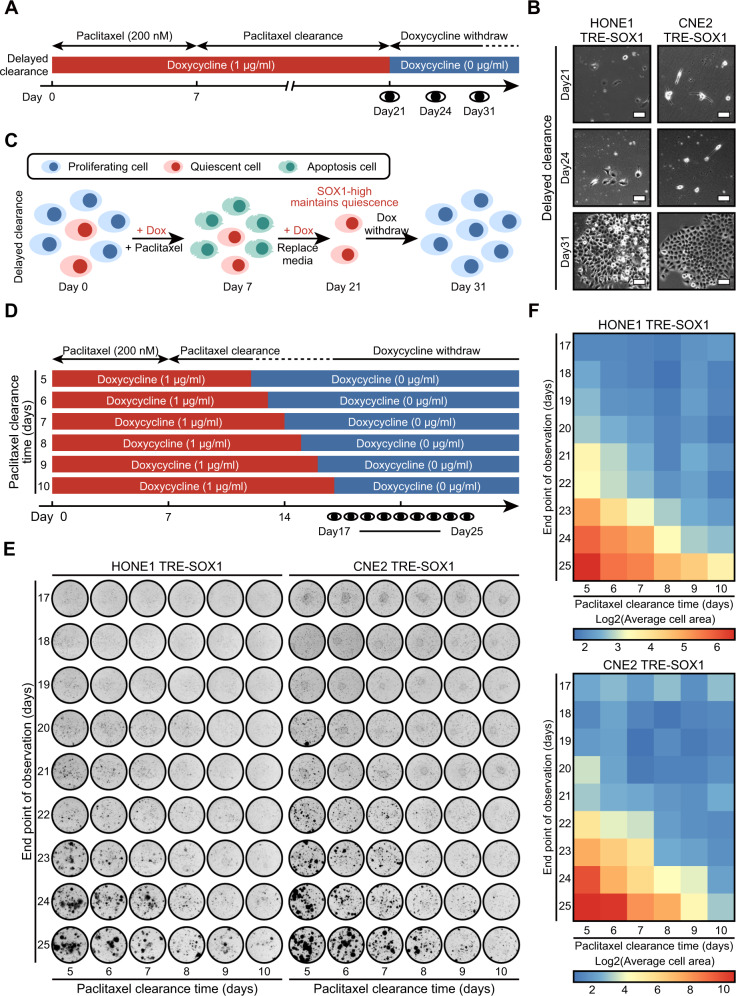
Decreased SOX1 expression determines the reactivation of QCCs. **A** An experimental timeline for multiple schedules of paclitaxel/doxycycline treatment in cells (HONE1 TRE-SOX1 or CNE2 TRE-SOX1). Cells were treated with doxycycline (Blue block: 0 µg/ml, red block: 1 µg/ml) to control SOX1 expression. Cells were pre-treated with doxycycline for 4 days and terminated on day 21. Meanwhile, cells were treated with 200 nM paclitaxel for 7 days followed by culturing in a paclitaxel-free environment. **B** Bright field images of the cells on day 21, day 24, and day 31. Scale bar = 50 µm. **C** An illustration of the model mimicking SOX1-induced QCCs. The “delayed clearance” group cells underwent killing of proliferative cells (day 0–7), clearance of intracellular paclitaxel (day 7–21), and reactivation of QCCs (day 21–31). Dox Doxycycline. **D** An experimental timeline for multiple schedules of paclitaxel/doxycycline treatment in cells (HONE1 TRE-SOX1 or CNE2 TRE-SOX1). Cells were treated with doxycycline (Blue block: 0 µg/ml, red block: 1 µg/ml) to control SOX1 expression. Cells were pre-treated with doxycycline for 4 days and terminated on day 12–day 17. Meanwhile, cells were treated with 200 nM paclitaxel for 7 days followed by culturing in a paclitaxel-free environment. **E** Colony formation assays on day 17–day 25 after treatment. **F** Heatmap showing the average cell sizes (log2 transformation) in colony formation assays.

We extended the duration of the “clearance of intracellular paclitaxel” stage from 7 to 14 days, during which the cells maintained a high expression level of SOX1 (Fig. 4A). Clonal clustered cells were only observed if the “reactivation of QCCs” stage lasted more than 6 days (Fig. 4B). The decline in SOX1 expression was corresponded to the reactivation of NPC cells from quiescence (Fig. 4C).

Furthermore, we discontinued doxycycline treatment between day 12 and day 17 (Fig. 4D). Colony formation assays showed that clonal clusters emerged later with prolonged doxycycline treatment (Fig. 4E, F). Therefore, the decreasing levels of SOX1 in QCCs determined the switch from a quiescence to a proliferation state.

### QCCs lacks of growth advantage in the absence of chemotherapy

**Fig. 5:**
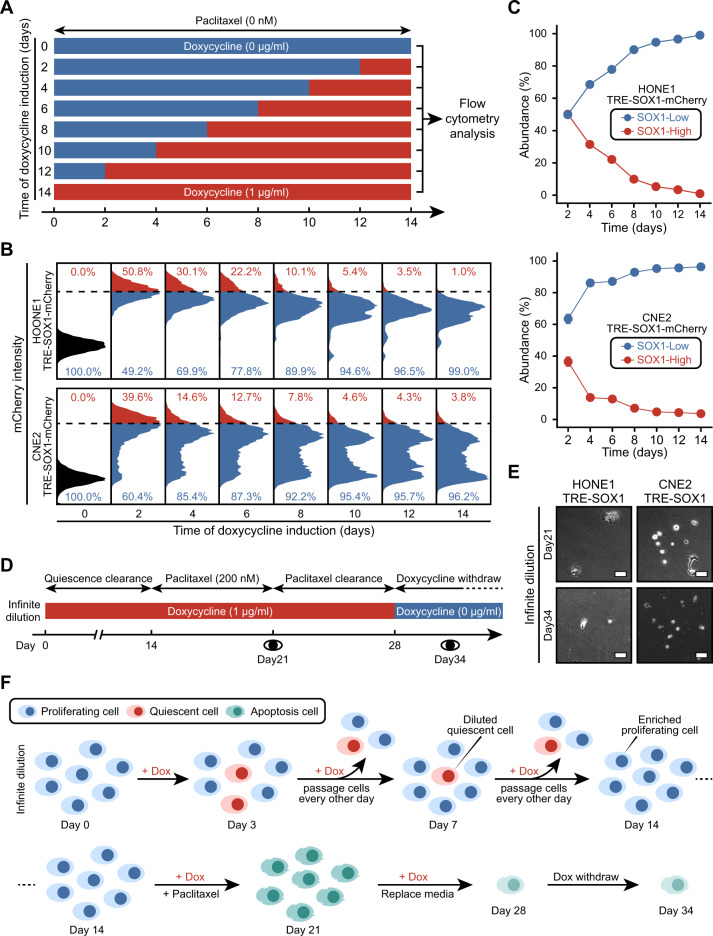
Growth advantage of cells with re-proliferation in the absence of paclitaxel treatment. **A** An experimental timeline for doxycycline (Blue block: 0 µg/ml, red block: 1 µg/ml) schedules used to control the expression of SOX1 in cells (HONE1 TRE-SOX1-mCherry or CNE2 TRE-SOX1-mCherry). On day 14, cells were collected for flow cytometry analysis. **B** Histograms showing the percentage of “SOX1-High” and “SOX1-Low” group cells. **C** Time courses of the abundance of “SOX1-High” and “SOX1-Low” group cells. **D** An experimental timeline for multiple schedules of paclitaxel/doxycycline treatment in cells (HONE1 TRE-SOX1 or CNE2 TRE-SOX1). The cells were treated with doxycycline (Blue block: 0 µg/ml, red block: 1 µg/ml) for 28 days to control SOX1 expression. Meanwhile, the cells were treated with 200 nM paclitaxel on day 14 and terminated on day 21. **E** Bright field images of the cells on day 21 and day 34. Scale bar = 50 µm. **F** An illustration of the model mimicking SOX1-induced QCCs. The “Infinite dilution” group cells underwent dilution of quiescent cells (day 0–14), killing of proliferative cells (day 14–21), clearance of intracellular paclitaxel (day 21–28), and reactivation of QCCs (day 28–34). Dox Doxycycline.

The growth benefits of the SOX1-induced QCCs were not apparent prior to the administration of paclitaxel. During prolonged culture, some of the quiescent cells were expected to disappear as the proliferative cells continued to grow and proliferate. Since not all cells were in a quiescent state and were still growing, we introduced a fusion of SOX1 with mCherry fluorescent protein into the cells and continued passaging to avoid contact inhibition of the cells (Fig. 5A). Flow cytometric analysis showed that the proportion of cells with high SOX1 expression, as indicated by mCherry fluorescence, gradually decreased over time and was <5% by day 14 (Fig. 5B, C). We speculated that the SOX1-induced QCCs were diluted during passaging. To address this, we pre-treated NPC cells with doxycycline for 2 weeks before administering paclitaxel (Fig. 5D). Interestingly, the morphology of the paclitaxel-treated cells exhibited either spherical (G2/M arrest) or giant (cytokinesis failure) phenotypes on day 21 (Fig. 5E). Following the discontinuation of doxycycline, no cells reactivated on day 34 (Fig. 5E). This method of infinite dilution showed an evolutionary trade-off between proliferative and quiescent NPC cells (Fig. 5F). Unlike cancer stem cells with self-renewal ability to maintain their proportion in tumors, the SOX1-induced QCCs did not exhibit similar characteristics.

**Fig. 6:**
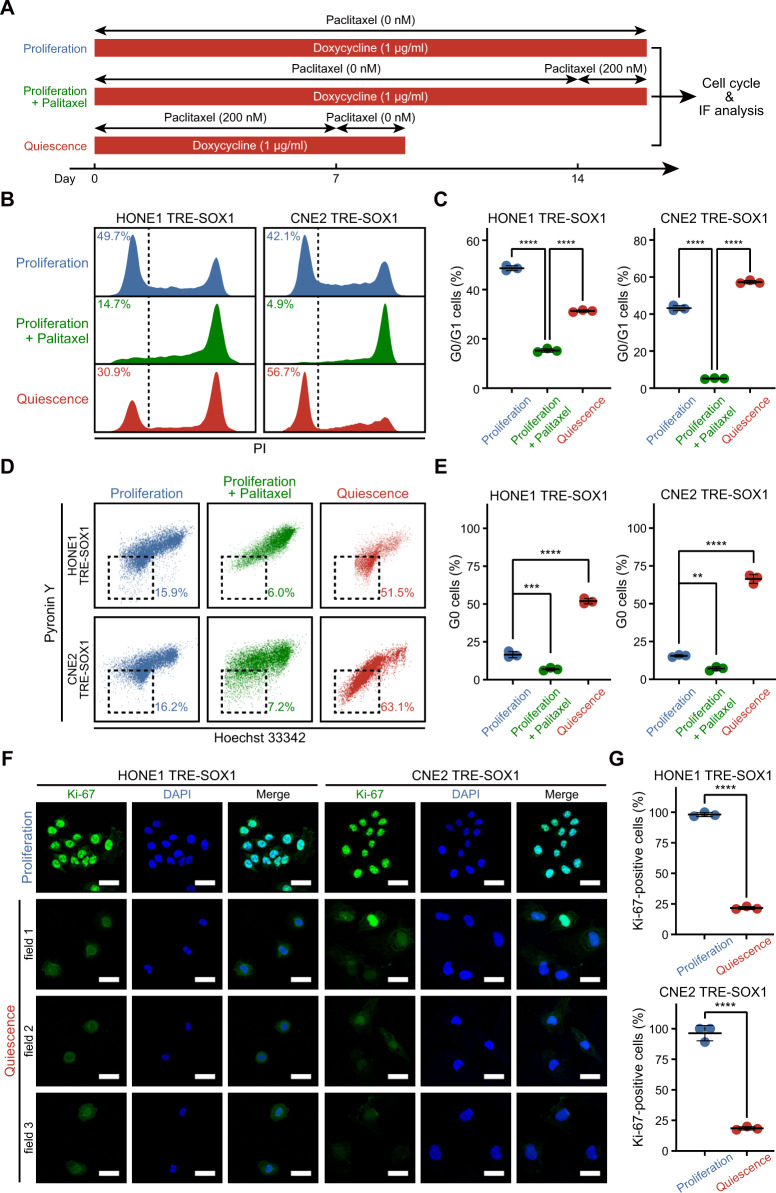
Characteristics of proliferative and quiescent NPC cells. **A** An experimental timeline for multiple schedules of paclitaxel/doxycycline treatment in cells (HONE1 TRE-SOX1 or CNE2 TRE-SOX1). Cells were treated with 1 µg/ml doxycycline (Red block) to overexpress SOX1. In the “Proliferation” or “Proliferation + Paclitaxel” group, cells were treated with doxycycline for more than 14 days. In the “Quiescence” group, cells were pre-treated with doxycycline for 4 days and terminated on day 9. Meanwhile, cells were treated with 200 nM paclitaxel during the indicated timeline. At the endpoint, cells were collected and used for cell cycle analysis or IF assays. **B** Histograms showing DNA contents (PI) in “Proliferation”, “Proliferation + Paclitaxel”, or “Quiescence” group of cells. The numbers represent the percentage of G0/G1 cells in total cells. **C** Scatter dot plots of the percentage of G0/G1 cells in cell cycle analysis. **D** Flow cytometric analysis of Hoechst 33342 and Pyronin Y staining in “Proliferation”, “Proliferation + Paclitaxel”, or “Quiescence” group of cells. Cells with DNA-Low/RNA-Low were gated as G0 cells. The numbers represent the percentage of G0 cells in total cells. **E** Scatter dot plots of the percentage of G0 cells in cell cycle analysis. **F** IF against Ki-67 in “Proliferation” and “Quiescence” groups of cells. Green: Ki-67, Blue: DAPI. Scale bar = 50 µm. **G** Scatter dot plots of the percentage of Ki-67-positive cells in IF assays.

To gain a better understanding of the distinction between the proliferative and quiescent subpopulations of NPC cells induced by SOX1, we conducted cell cycle analysis (Fig. 6A). After 2 days of treatment with paclitaxel, cells in the “Proliferation+Paclitaxel” group were mostly arrested in the G2/M phase, indicating that these cells had little G0 phase prior to drug treatment (Fig. 6B, C). In contrast, the “Quiescence” group showed a large proportion of cells that were still located in the G0/G1 phase even after 7 days of paclitaxel treatment, indicating that these cells possessed a significantly slower proliferation rate (Fig. 6B, C). Similarly, the “Quiescence” group contained more than 50% of cells with low DNA/RNA contents (in G0 phase) (Fig. 6D, E). Furthermore, the IF assay showed that most of the cells (~80%) in the “Quiescence” group were Ki-67-negative (Fig. 6F, G).

**Fig. 7:**
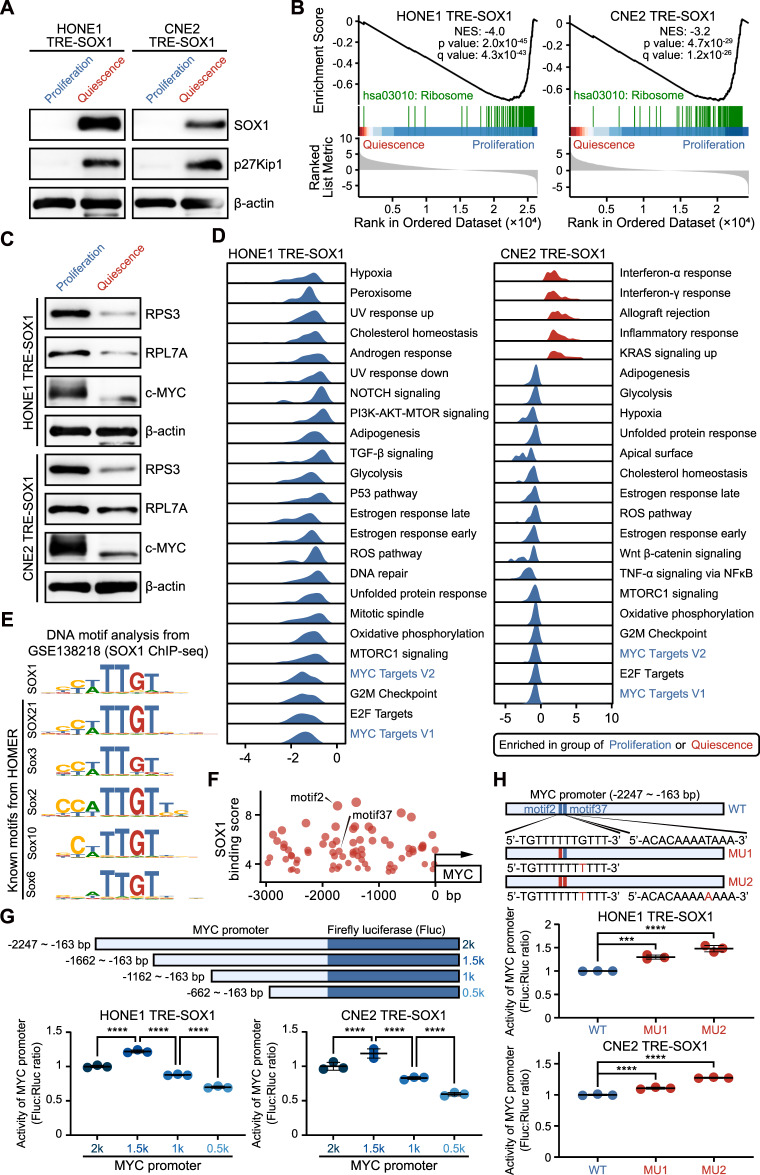
Down-regulated MYC signaling in SOX1-induced quiescent NPC cells. **A** Western blot analysis of SOX1, p27Kip1 and β-actin expression in “Proliferation” and “Quiescence” group of cells. β-actin was used as a control. **B** GSEA of ranked genes from cells. Gene set “hsa03010: Ribosome” of KEGG pathways was shown. NES normalized enrichment score. **C** Western blot analysis of RPS3, RPL7A, c-MYC and β-actin expression in “Proliferation” and “Quiescence” group of cells. β-actin was used as a control. **D** Ridgeplots showing MSigDB Hallmark gene sets based on the GSEA analysis of proliferative and quiescent NPC cells. The “Proliferation” (blue) or “Quiescence” group (red) of cells was enriched in the indicated gene sets. **E** Motifs analysis of SOX1-binding peaks. The top de novo motif for SOX1-binding is presented, along with several similar known motifs. **F** Matched sequences to SOX1-binding de novo motif in MYC promoter. A higher score indicates that the match is more significant. **G** Upper panel, truncated designs of different MYC promoters on a luciferase reporter vector. Lower panel, dual luciferase assay evaluating the effects of SOX1 on the truncated (2k, 1.5k, 1k, and 0.5k) MYC promoter reporter constructs. **H** Upper panel, mutant designs of different MYC promoters on a luciferase reporter vector. Lower panel, dual luciferase assay evaluating the effects of SOX1 on the wild-type (WT) or mutant (MU1 and MU2) MYC promoter reporter constructs.

We performed RNA-seq analysis to investigate the expression profiles of these two groups (Supplementary Fig. S6A). The PCA analysis showed that the within-group distributions of gene expression profiles were well-clustered (Supplementary Fig. S6B). The “Proliferation” group was dominated by cells with low expression of SOX1, while the “Quiescence” group exhibited high expression of p27Kip1, an established marker for quiescent cells (Fig. 7A, Supplementary Fig. S6C). GSEA showed a significant decrease in enrichment of the ribosome pathway in “Quiescence” group cells (Fig. 7B, Supplementary Fig. S6D), and western blot analysis verified the reduced expression of RPS3 and RPL7A (Fig. 7C). In addition, the gene sets associated with cell cycle, DNA replication, and metabolic and biosynthetic processes, were also significantly downregulated (Supplementary Tables S4, S5). The hallmark gene sets associated with several cell growth signaling pathways were significantly de-enriched in “Quiescence” group cells, such as “MYC Targets V1”, “MYC Targets V2”, “E2F Targets”, “G2M Checkpoint”, “MTORC1 signaling”, and “Oxidative phosphorylation” (Fig. 7D, Supplementary Tables S6, S7).

### SOX1 downregulates MYC and induces QCCs through its transcriptional function

c-MYC (MYC) was identified as a master regulator of ribosome synthesis [18], with its signaling pathway showing the most significant enrichment in GSEA analysis based on differential expressed genes between “Proliferation” and “Quiescence” group cells (Supplementary Tables S6, S7). Notably, the ribosome pathway also showed prominent enrichment in “Proliferation” group (Supplementary Tables S4, S5). Therefore, SOX1 might suppress the ribosome synthesis by downregulating MYC levels. Western blot analysis confirmed that MYC protein levels were low in the “Quiescence” group cells (Fig. 7C). As a transcription factor, MYC plays a crucial role in regulating cell cycle and cell growth. Therefore, we were interested in how SOX1 regulated the levels of MYC. The SOX family proteins share a conserved high-mobility group (HMG) domain, binding to the same core consensus DNA motif (5’-TTGT) [19]. We analyzed de novo and known motifs in SOX1 peaks from the GEO database (Fig. 7E, Supplementary Table S8). The promoter of the *MYC* gene contained many potential SOX1-binding sequences (Fig. 7F, Supplementary Table S9). Thus, SOX1 might downregulate *MYC* transcription by binding to the *MYC* promoter and recruiting transcriptional repressors. To investigate this mechanism, we designed luciferase reporter vectors with different lengths of truncated *MYC* promoter and showed that deletion in the -2247 − -1662 bp region led to an increase in transcriptional levels (Fig. 7G), suggesting that SOX1 might play a major inhibitory role by binding in this region. To further explore this, we mutated two SOX1-binding DNA motifs in this region and observed an increase in transcriptional levels in the mutated *MYC* promoter (Fig. 7H). The evidence suggests that SOX1 exerts a transcriptional repression effect by binding to the *MYC* promoter.

**Fig. 8:**
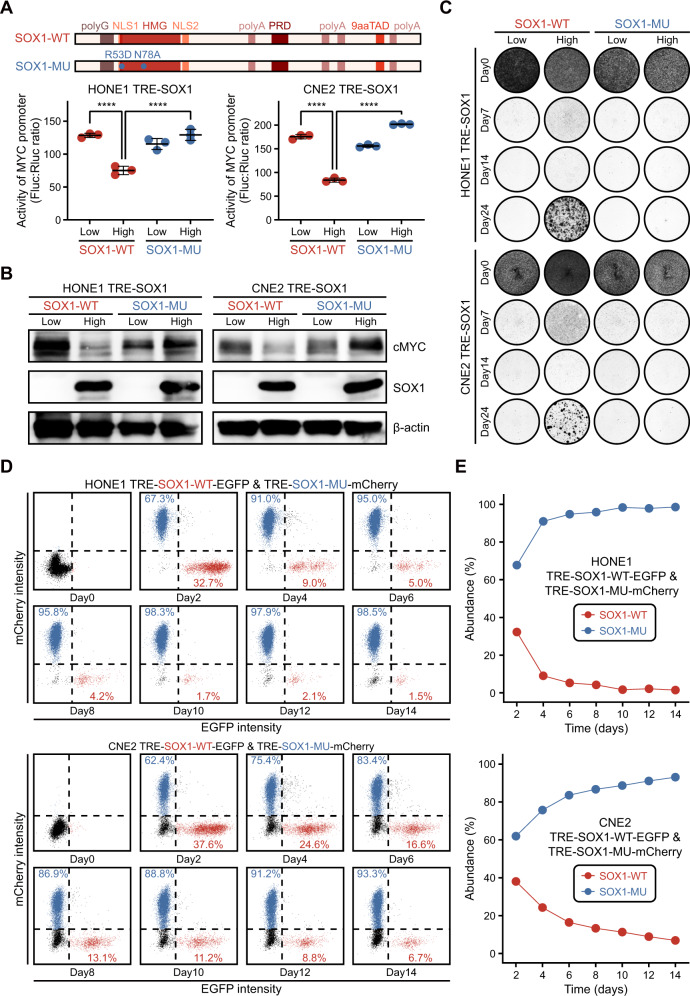
Proliferation and quiescence state orchestrated by SOX1-MYC. **A** Upper panel, schematic of wild-type (WT) and mutant (MU) SOX1 used in this study. Lower panel, dual luciferase assay evaluating the effects of WT and MU SOX1 on the MYC promoter reporter construct. **B** Western blot analysis of c-MYC, SOX1 and β-actin expression in cells expressing WT or MU SOX1. β-actin was used as a control. **C** Colony formation assay of cells with overexpression of WT or MU SOX1 on day 24, following the experimental timeline in Fig. 2A. **D** Two stable cell lines (HONE1 TRE-SOX1-WT-EGFP:HONE1 TRE-SOX1-MU-mCherry or CNE2 TRE-SOX1-WT-EGFP:CNE2 TRE-SOX1-MU-mCherry) were mixed in a 1:1 ratio. Dot plots showing the percentage of cells expressing WT or MU SOX1 within populations of single positive cells (mCherry single positive and EGFP single positive), following the experimental timeline in Fig. 5A. **E** Time courses of the abundance of cells expressing WT or MU SOX1 within populations of single positive cells (mCherry single positive and EGFP single positive).

In the GSEA analysis, we observed downregulation of several cell proliferation-related pathways in “Quiescence” group cells (Supplementary Tables S6, S7). Considering that SOX1 is a transcription factor, we hypothesized that SOX1 might exert its role in inducing cell quiescence through regulation of downstream genes expression via transcriptional function. We mutated two amino acids (Arg53 to Asp and Asn78 to Ala) in HMG domain of SOX1 to abolish its transcriptional function, as previously verified in our work [16]. The luciferase reporter system confirmed that the mutated SOX1 was incapable of suppressing the transcriptional levels driven by the MYC promoter (Fig. 8A), and this was also observed at the protein level (Fig. 8B). In addition, the formation of colony was completely inhibited in the mutant group (Fig. 8C), suggesting that SOX1 induced cell quiescence through its transcriptional function. Finally, we constructed stable cell lines expressing wild-type SOX1 fused with EGFP and mutant SOX1 fused with mCherry, respectively. These cell lines were mixed in a 1:1 ratio and induced with doxycycline to observe the impact of the two types of SOX1 on cell growth advantage. The cells with mutant SOX1 had gradually outcompeted those with wild-type SOX1, occupying the majority of the cell population within a few days (Fig. 8D, E). This further supports the finding that tumor cells with high expression of SOX1 lack growth advantage in a stress-free environment.

## Discussion

**Fig. 9:**
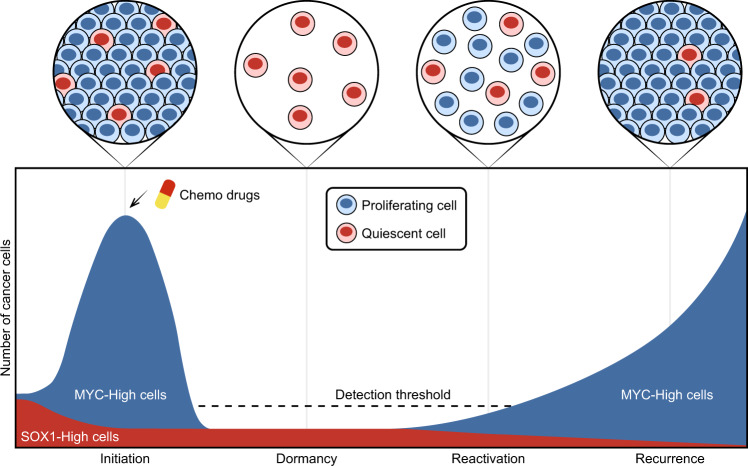
Schematic representation of evolutionary trade-off between proliferative and quiescent NPC cells orchestrated by oncogene and tumor hypnotist. The SOX1-induced QCC models mimic the four main stages of cancer development (initiation, dormancy, reactivation, and recurrence). In the stage of initiation, cancer cells are highly proliferative due to the action of oncogene (MYC). After chemotherapy, the dominant cell populations switch from proliferative to quiescent cells. In the stage of dormancy, high expression of tumor hypnotist (SOX1) in QCCs maintains a long-term cellular dormancy. Detecting these latent QCCs using conventional clinical testing methods is challenging. In the stage of reactivation, QCCs with decreased SOX1 levels recover with re-expression of MYC. In the stage of recurrence, MYC-driven cancer cells lead to a relapsed tumor, reaching the detection threshold.

In general, patients with high expression levels of tumor suppressors have favorable prognosis. Loss-of-function mutations in tumor suppressors, such as RB1, TP53, PTEN, and CDKN2A, contribute to cancer progression and poor prognosis. However, low frequency of genetic alterations is observed in SOX1, suggesting that this tumor suppressor has a secondary mechanism for unfavorable prognosis. Our study presents that overexpression of SOX1 can induce quiescence in individual NPC cells, implying that the quiescent NPC cells are resistant to chemotherapy. Similarly, the tumor suppressor p21Cip1 has also been reported to be correlated with poor prognosis in some cancer types [20]. Unlike that tumor suppressors inhibit cell proliferation and tumor development, “tumor hypnotists” promote cancer cells to enter a quiescent state. Our model describes a dynamic process in vitro that mimics cancer development going through stages of initiation, dormancy, reactivation, and recurrence (Fig. 9). In the stage of initiation, cancer cells with low levels of SOX1 have a growth advantage within the population, while those with high levels of SOX1 facilitate cancer cell survival against treatment of chemo drugs. During dormancy, high expression of SOX1 maintains the long-term quiescent state, where the limited size of tumors makes it difficult for detection. In the stage of reactivation, some QCCs may decrease SOX1 and re-express MYC protein, resulting in increased abundance of proliferative cancer cells. Eventually, tumor relapse would be observed when the number of cancer cells were above the detectable threshold.

Our experimental findings indicate that SOX1-induced QCCs might pose a potential threat in a clinical setting. The chemo drugs not only kill fast-growing cancer cells but also normal cycling cells, leading to side effects. SOX1-induced QCCs could survive for over 2 weeks after treatment of high doses of paclitaxel. The chemo drugs may prefer to kill normal cells before targeting QCCs due to a slower growth rate, ultimately making these drugs less effective. In addition, scattered individual QCCs are challenging to be detected using current clinical testing methods. Our model showed that high levels of SOX1 could maintain the quiescent state for at least 2 weeks without forming clustered colonies. The decreased SOX1 levels determined when QCCs converted to a proliferative state, suggesting that the re-activation of these cells would have possibility to be controlled in a way of perturbation in SOX1 expression.

Although SOX1 is usually expressed at low levels in several tumors, a small subset of QCCs with high expression of SOX1 play an essential role in drug-resistance. The presence of a few SOX1-positive cancer cells within tumor lesions might indicate that traditional chemotherapy fails to eliminate all cancer cells, even if the patient has achieved a complete response. Thus, further studies are needed to investigate the potential of SOX1 as a prognostic biomarker for residual NPC cells and its association with treatment response in patients. Meanwhile, we suggest that detection of SOX1 levels at the single-cell resolution using clinical methods such as immunohistochemistry or single-cell sequencing, is essential as bulk tumors have high heterogeneity.

Currently, three main strategies are being explored to target QCCs in tumors, including (i) reactivating QCCs and targeting them with cell-cycle-dependent anti-cancer agents, (ii) maintaining cells in a quiescent state, and (iii) targeting the unique features of QCCs [21]. For the first strategy, a combination of chemotherapy and drugs that inhibit SOX1 levels could be considered. However, targeting SOX1, a transcription factor, is challenging due to the limited availability of inhibitors. A study has developed small-molecule inhibitors for the HMG DNA-binding domain of SOX18 [22], which may provide a potential way to interfere with the HMG box domain in SOX1 since the mutated HMG box disrupt the switch from proliferation to quiescence. For the second strategy, methods to maintain high SOX1 expression in QCCs could be explored. However, due to the limitations of the artificial gene expression model, it remains unknown how SOX1 is induced in individual cancer cells to enter quiescence or how QCCs decrease SOX1 expression to resume proliferation in response to environmental alterations. The last strategy involves targeting SOX1-positive cancer cells directly by developing antibody-drug or small molecule-drug conjugates to eliminate QCCs. This approach has the potential to specifically target the unique features of QCCs and improve treatment outcomes. However, further investigations are needed to develop effective drugs and optimize drug delivery to ensure the efficacy against QCCs.

The best therapeutic approach is still unknown due to the development of resistance. Our study provides a model that mimics QCCs induced by intrinsic factors. Unlike other in vitro models, our model is independent of extracellular cell growth signaling, providing a new method to deepen our understanding of the initiation or termination of quiescence in cancer. This could also help us understand the molecular mechanisms of SOX1 in different stages of cancer progression.

## Conclusion

We suggest to re-classify the intrinsic transcriptional factor SOX1 as a “tumor hypnotist” rather than a “tumor suppressor”, based on its dynamic roles in tumor development. The high expression of SOX1 within individual cancer cells could potentially indicate chemotherapy resistance in NPC, and it might be of significance to propose feasible targeting strategies for tumor recurrence after clinical interventions.

## Materials and methods

### In silico analysis

Datasets of RNA expression for various human cancer types were obtained from the pan-cancer atlas, which is based on the TCGA (The Cancer Genome Atlas) database (https://gdc.cancer.gov/about-data/publications/pancanatlas). To explore the mutation characteristics of SOX1 and clinicopathological features in different tumors, the cBioPortal database was utilized (https://www.cbioportal.org) [23]. Survival analysis was conducted using the online website tool GEPIA (Gene Expression Profiling Interactive Analysis, http://gepia.cancer-pku.cn/index.html) [24]. The expression values of SOX1 were extracted and visualized in a violin plot created using GraphPad Prism software (version 9.0.0).

### Compounds and antibodies

Doxycycline (HY-N0565B) and CFSE (HY-D0938) were purchased from Medchem Express (MCE). Bovine serum albumin (BSA, FC0077) and propidium iodide (PI, 219545810) were purchased from MP Biomedicals. RNase A (10406ES03) was purchased from Yeasen Biotechnology. 5-Bromo-2′-deoxyuridine (BrdU, B5002), Hoechst 33342 (B2261), Triton X-100 (T8787), Pyronin Y (213519), and 4′,6-Diamidino-2-phenylindole dihydrochloride (DAPI, D9542) were purchased from Sigma-Aldrich. Paclitaxel was purchased from Beijing SL Pharmaceutical Co., Ltd. (China). Cisplatin was purchased from Jiangsu Hansoh Pharmaceutical Co., Ltd. (China).

Antibodies against SOX1 (ab109290) and RPS3 (ab128995) were purchased from Abcam. Antibody against RPL7A (DF9137) was purchased from Affinity Biosciences. Antibodies against β-actin (60008-1-Ig), Ki-67 (27309-1-AP), and c-MYC (10828-1-AP) were purchased from Proteintech. Antibody against p27 Kip1 (sc-528) was purchased from Santa Cruz Biotechnology. Alexa Fluor 488 cross-adsorbed goat anti-rabbit IgG secondary antibody (A11008) was purchased from Invitrogen.

### Plasmid constructs

Plasmids that encode human SOX1 and its mutant were created using PCR amplification and subcloning into the pLVX-TRE3G expression vector through the ClonExpress II One Step Cloning Kit or ClonExpress MultiS One Step Cloning Kit (Vazyme, C112-02 or C113-02), following the manufacturer’s instructions. The plasmid was named “pLVX-TRE-SOX1”. The primers utilized for gene cloning can be found in our previous work [16].

The human MYC promoter, as well as its mutant and truncated forms, were created through PCR amplification and subcloning into the pGL6 reporter vector (Beyotime, D2102) using either the ClonExpress II One Step Cloning Kit or ClonExpress MultiS One Step Cloning Kit. The primers utilized for luciferase reporter are listed in Supplementary Table S10.

### Lentiviral production, infections, and cell line generation

Lentivirus was produced by transient transfection by Lipofectamine 2000 (Invitrogen, 11668019) in human embryonic kidney (HEK) 293 T cells utilizing a second-generation lentiviral vector system (psPAX2 and pMD2.G). All virus-containing medium was mixed with 8 μg/ml polybrene (Sigma-Aldrich, H9268). The viruses that resulted from the pLVX-Tet3G plasmid were utilized to infect wild-type CNE2 or HONE1 cells in order to establish HONE1-Tet-On or CNE2-Tet-On stable cell lines. Cells were selected in 1 mg/ml G418 for at least 2 weeks. Following this, HONE1-Tet-On and CNE2-Tet-On cell lines were infected with viruses that were produced from pLVX-TRE-SOX1 plasmids and selected using 2 μg/ml puromycin for 6 days.

### Cell culture and SOX1 induction

The HONE1 and CNE2 cell lines were obtained from Dr. Chao-Nan Qian (Sun Yat-sen University, Guangzhou, China). Wild-type cell lines and their lentiviral-infected stable cell lines were all maintained in RPMI 1640 (Gibco, 11875119) supplemented with 10% fetal bovine serum (HyClone, SH30070.03). The cells were incubated at 37 °C in a humidified chamber containing 5% CO_2_. All cells were routinely tested for mycoplasma.

The HONE1 TRE-SOX1 and CNE2 TRE-SOX1 cell lines were treated with 1 μg/ml doxycycline in order to induce overexpression of SOX1. The decrease in SOX1 expression was controlled by renewing the culture medium with no doxycycline.

### RNA-sequencing (RNA-Seq) and pre-processed data

The total RNA was extracted from cells using HiPure total RNA Mini Kit (Magen, R4111-03) according to the manufacturer’s instructions. The RNA-Seq data was generated and normalized using an Illumina HiSeq™ PE150 system by Novogene Bioinformatics Technology Co., Ltd. (Beijing, China). The raw data was processed using “trim_galore”, “hisat2”, “samtools”, and “featureCounts” software on a Linux system. RNA-Seq expression datasets were loaded into R software (version 4.1.0) for principal component analysis (PCA) or differential expression analysis using the “DESeq2” package. The volcano diagrams were created using the “ggplot2” package.

### Gene set enrichment analysis (GSEA)

GSEA was carried out using gene sets from the KEGG Pathway Database (https://www.genome.jp/kegg/pathway.html) or Hallmark gene sets from the Human Molecular Signatures Database (MSigDB, https://www.gsea-msigdb.org/gsea/msigdb/ human/collections.jsp). The ranked genes based on fold change were analyzed using the “gseKEGG” function in the “clusterProfiler” package [25] in R software (version 4.1.0). The output was a list of gene set ranks containing information on normalized enrichment scores (NES), *P* value, and FDR-q-value. The selected gene sets were visualized using “gseaplot2” and “ridgeplot” functions in the “clusterProfiler” package to create GSEA and ridge plots, respectively. The KEGG pathway graphs were generated using the “pathview” package.

### Cell lysis, protein concentration and Western blot (WB) analysis

Cells were digested, counted, and lysed on ice in RIPA buffer (Beyotime, P0013B) containing a cocktail of protease inhibitors (TargetMol, C0001). Protein concentration was determined using the Bradford protein assay, and BSA was used as the standard substance. The protein content per cell was calculated by combining the data from cell counts and protein concentration. Equal amounts of cell extracts were subjected to electrophoresis in 10% SDS-PAGE gels and transferred onto 0.45 µm PVDF membranes (Millipore, IPVH00010) for antibody blotting. A horseradish peroxidase-conjugated goat anti-mouse or goat anti-rabbit IgG (Pierce, 31430 or 31460) was used as the secondary antibody. The proteins were visualized using an Omni-EC™ enhanced pico light chemiluminescence kit (EpiZyme, SQ101) and imaged using a ChemiDoc MP imager (Bio-Rad). The original western blot images have been provided in the Supplemental Material files.

### BrdU incorporation assay and flow cytometric analysis

All cell samples were seeded into six-well tissue culture plates (Jet Biofil, TCP010006) on the same day and incubated for 4 days. To prevent cell-cell-contact-induced inhibition of cell proliferation, cells were passaged to a new 6-well plate at a cell density of 20% confluency on day 2. BrdU labeling and staining were carried out using the FITC-BrdU cell proliferation detection kit (Keygen Biotech, KGA319-1) following the manufacturer’s instructions. The percentage of BrdU-positive cells (FITC: ex 494 nm/em 520 nm) was analyzed by flow cytometry (CytoFLEX, Beckman Coulter).

### Colony formation assay

Cells were initially seeded into six-well or 12-well tissue culture plates (Jet Biofil, TCP010012) at a density of 2 × 10^5^ or 1 × 10^5^ cells/well, respectively. The “SOX1-High” group cells were pre-treated with 1 μg/ml doxycycline for 4 days. After the incubation period, the culture medium was removed, and the cells were fixed with 4% paraformaldehyde (Biosharp, BL539A) for 25 min and stained with crystal violet staining solution (Beyotime, C0121) for 30 min. Following this, the cells were gently washed and air-dried. The plates were then imaged using a ChemiDoc MP imager (Bio-Rad), and the average sizes of the clone clusters were calculated using ImageJ software (version 1.53c). The heatmaps of the log2-transformed average sizes were generated using the “pheatmap” library in R software (version 4.1.0).

### 5-ethynyl-2′-deoxyuridine (EdU) incorporation assay and Ki-67 staining

Cells were initially seeded into 96-well tissue culture plates (Jet Biofil, TCP011096) at a density of 5 × 10^3^ cells/well. The “SOX1-Low” group cells were passaged every other day, while the “SOX1-High” group cells were pre-treated with 1 μg/ml doxycycline for 4 days, followed by additional paclitaxel treatment for 7 days. On day 6, EdU solution was added to each well, and after 24 h, EdU staining was performed using Kfluor488-EdU cell proliferation kit (Keygen Biotech, KGA331-100) according to the manufacturer’s instructions. The cells were then incubated with the Ki-67 antibody (dilution 1:200) at 4 °C overnight. The immune complexes were stained with the secondary antibody conjugated to Alexa-546 (dilution 1:200) at RT for 1 h. The nuclei were counterstained with DAPI and viewed using a Nikon ECLIPSE Ti2 microscope (DAPI: ex 358 nm/em 461 nm; kFluor488-azide: ex 495 nm/em 520 nm; Alexa Fluor 546: ex 556 nm/em 573 nm). The percentage of EdU and Ki-67 double negative cells in total was determined by analyzing the images with ImageJ software (version 1.53c).

### Carboxyfluorescein diacetate succinimidyl ester (CFSE) proliferation assay and flow cytometric analysis

The “SOX1-High” group cells were pre-treated with 1 μg/ml doxycycline for 4 days. The cells were resuspended and seeded onto six-well tissue culture plates or 10 cm tissue culture dishes (Jet Biofil, TCD010100) at an initial density of 2 × 10^5^ cells/well or 5 × 10^6^ cells/dish, respectively. Next, the cells were incubated with 5 µM CFSE staining solution at 37 °C for 10 min. The “SOX1-Low” group cells were passaged every other day. On day 7, flow cytometry (CytoFLEX, Beckman Coulter) was used to measure the intensity of CFSE (CFSE: ex 495 nm/em 520 nm). The mean fluorescence intensity (MFI) of CFSE was calculated using FlowJo software (version 10.5.3).

### Cell cycle analysis

The cells were pre-treated with 1 μg/ml doxycycline for 4 days before being seeded onto 10 cm tissue culture dishes at an initial density of 5 × 10^6^ cells/dish. The cells were then digested, washed, and fixed with 70% cold ethanol at 4 °C overnight. To analyze the cell cycle distribution, the nuclei of cells were labeled by PI staining solution (0.2% Triton X-100, 100 μg/ml RNase A, and 50 μg/ml PI in PBS) at room temperature (RT) for 30 min. Subsequently, flow cytometry (CytoFLEX, Beckman Coulter) was used to analyze the DNA content of the cells (PI: ex 535 nm/em 615 nm). For labeling G0 cells, the cells were resuspended in PBS containing 10 µg/ml Hoechst 33342 and incubated at 37 °C for 45 min. Pyronin Y was then added to the cells at a final concentration of 5 µg/ml, and the cells were incubated at 37 °C for another 45 min. Finally, flow cytometry (CytoFLEX, Beckman Coulter) was used to analyze the G0 cells (Hoechst 33342: ex 350 nm/em 460 nm; Pyronin Y: ex 546 nm/em 565 nm). The proportion of G0/G1 cells was calculated using FlowJo software (version 10.5.3).

### Immunofluorescence (IF) staining

The cells were fixed in 4% paraformaldehyde at RT for 20 min and then permeabilized in 0.5% Triton X-100 in PBS for 5 min. The slides were then incubated with the primary antibody (dilution 1:200) at 4 °C overnight. The immune complexes were stained with the secondary antibody conjugated to Alexa-488 (dilution 1:200) at RT for 1 h. The nuclei were counterstained with DAPI and viewed using a Nikon ECLIPSE Ti2 microscope (DAPI: ex 358 nm/em 461 nm; Alexa Fluor 488: ex 495 nm/em 519 nm).

### Data analysis of chromatin immunoprecipitation sequencing (ChIP-Seq)

The raw data were obtained from the GEO database (GSE138218), and two samples (GSM4102054 and GSM4102055) were processed using various software in a Linux system, including “fastq-dump”, “bowtie2”, “samtools”, “macs2”, and “HOMER”. The SOX1-binding de novo motif was uploaded to the FIMO website tool (MEME Suite, version 5.5.1), and the software was used to analyze sequences that matched the promoter of the MYC gene (https://meme-suite.org/meme/tools/fimo) [26].

### Luciferase reporter assay

The cells were pre-treated with 1 μg/ml doxycycline for 1 day. Afterwards, the cells were seeded onto six-well tissue culture plates at a density of 4 × 10^5^ cells/well. The cells were co-transfected with both the wild-type and mutated MYC promoter reporter plasmid and pRL-TK plasmids. To measure the luciferase activity, the Dual Luciferase Reporter Assay System (Promega, E1910) was used 24–48 h after transfection.

### Statistical analysis

Each experiment was performed at least three biological replicates and results were presented as recorded values from each sample with arithmetic means ± standard deviation (SD), unless otherwise indicated. Statistical analysis was conducted using GraphPad Prism software (version 9.0.0). Appropriate tests such as chi-squared tests, Fisher’s exact tests, two-tailed Student’s *t* tests, or one-/two-way ANOVA tests with multiple comparisons were used. Before conducting these tests, homogeneity of variance was checked. A *P* value < 0.05 was considered statistically significant (n.s.: not significant, *: *p* value < 0.05, **: *p* value < 0.01, ***: *p* value < 0.001, ****: *p* value < 0.0001).

## Supplementary information

Supplemental Figures Legends

Supplementary Figure S1

Supplementary Figure S2

Supplementary Figure S3

Supplementary Figure S4

Supplementary Figure S5

Supplementary Figure S6

Supplementary Table S1

Supplementary Table S2

Supplementary Table S3

Supplementary Table S4

Supplementary Table S5

Supplementary Table S6

Supplementary Table S7

Supplementary Table S8

Supplementary Table S9

Supplementary Table S10

Original western blots

## Data Availability

All supporting data are included in the paper and supplemental files. Additional data are available upon reasonable request to the corresponding author.
