# Supplementary material for: SOX1 acts as a tumor hypnotist rendering nasopharyngeal carcinoma cells refractory to chemotherapy
Source: Cell Death Discov. 2023 Jun 27;9:194. doi: 10.1038/s41420-023-01479-x (PMC10300072; doi:10.1038/s41420-023-01479-x)
Supplement: Supplementary file 1 — Supplemental Figures Legends [file 41420_2023_1479_MOESM1_ESM.docx]

## Supplementary Figure S1 Expression and genetic alteration of SOX1 across various human cancers.

**(A)** Violin plot illustrating the mRNA expression level of *SOX1* in various cancer tissues obtained from the TCGA database. **(B)** The alteration frequency with mutation types of *SOX1* in different tumors from the TCGA database. (THCA, thyroid cancer; BRCA, breast invasive carcinoma; OV, ovarian serous cystadenocarcinoma; LIHC, liver hepatocellular carcinoma; UCEC, uterine corpus endometrial carcinoma; PRAD, prostate adenocarcinoma; CESC, cervical squamous cell carcinoma and endocervical adenocarcinoma; PAAD, pancreatic adenocarcinoma; KICH, kidney chromophobe; KIRC, kidney renal clear cell carcinoma; KIRP, kidney renal papillary cell carcinoma; BLCA, bladder urothelial carcinoma; LUAD, lung adenocarcinoma; LUSC, lung squamous cell carcinoma; TGCT, testicular germ cell tumors; SKCM, skin cutaneous melanoma; HNSC, head and neck squamous cell carcinoma; COAD, colon adenocarcinoma; READ, rectum adenocarcinoma; GBM, glioblastoma multiforme; STAD, stomach adenocarcinoma; SARC, sarcoma; UCS, uterine carcinosarcoma; ESCA, esophageal carcinoma; MESO, mesothelioma; ACC, adrenocortical carcinoma; DLBC, lymphoid neoplasm diffuse large B-cell lymphoma; LGG, brain lower grade glioma; PCPG, pheochromocytoma and Paraganglioma; LAML, acute myeloid leukemia; CHOL, cholangiocarcinoma; UVM, uveal melanoma; THYM, thymoma)

## Supplementary Figure S2 Clinical prognosis of individual patients with various human cancers stratified by SOX1 expression.

**(A&B)** Kaplan-Meier survival curves showing the differences in overall survival (**A**) or disease-free survival (**B**) between high and low expression groups stratified by SOX1 for patients from the TCGA database. (Log-rank test) **(C)** Violin plots illustrating the distribution of SOX1 mRNA expression levels in cancer tissues from the TCGA database, stratified by the occurrence of "New neoplasm event post initial therapy". **(D&E)** Kaplan-Meier survival curves showing the differences in overall survival (**D**) or disease-free survival (**E**) between high and low expression groups stratified by SOX1 in patients with early-stage or late-stage HNSC from the TCGA database. (Log-rank test) (HNSC, head and neck squamous cell carcinoma; UVM, uveal melanoma; SKCM, skin cutaneous melanoma; COAD, colon adenocarcinoma; READ, rectum adenocarcinoma; TGCT, testicular germ cell tumors; GBM, glioblastoma multiforme; LGG, brain lower grade glioma)

## Supplementary Figure S3 RNA-seq analysis of NPC cells with high versus low SOX1 expression.

**(A)** An experimental timeline for doxycycline (Blue block: 0 µg/ml, red block: 1 µg/ml) schedules used to control the expression of SOX1 in cells (HONE1 TRE-SOX1 or CNE2 TRE-SOX1). On day 3, cells were harvested and total RNA was extracted for RNA-seq analysis. **(B)** Principal component analysis (PCA) of gene expression profiles from cells (HONE1 TRE-SOX1 or CNE2 TRE-SOX1) with high versus low SOX1 expression. **(C)** Volcano diagram of the differentially expressed genes (DEGs) between cells (HONE1 TRE-SOX1 or CNE2 TRE-SOX1) with high versus low SOX1 expression. SOX1 is highlighted. **(D)** KEGG pathway maps showing up- (red) or down-regulated (blue) genes in ribosome pathway. Each cell represents the log2 ratio gene expression data of the “SOX1-High” group normalized to the “SOX1-Low” group in NPC cells (HONE1 TRE-SOX1 or CNE2 TRE-SOX1).

## Supplementary Figure S4 Wild type NPC cells under cell-cycle specific chemotherapy.

Wild type NPC cells (HONE1 or CNE2) were treated with various concentrations of paclitaxel (0, 20, 50, 100, 200, or 500 nM) for 72 hours and observed under a bright field light microscope. Scale bar = 50 µm.

## Supplementary Figure S5 A model mimicking SOX1-induced QCCs under cell cycle-nonspecific chemotherapy.

**(A)** An experimental timeline for multiple schedules of cisplatin/doxycycline treatment in cells (HONE1 TRE-SOX1 or CNE2 TRE-SOX1). The cells were treated with doxycycline (Blue block: 0 µg/ml, red block: 1 µg/ml) to control SOX1 expression. The “SOX1-High” group cells were pre-treated with doxycycline for 4 days and terminated on day 14. Both groups of cells were treated with 50 µM cisplatin for 7 days followed by culturing in a cisplatin-free environment. **(B)** Bright field images of the cells on day 3 and day 20. Scale bar = 50 µm.

## Supplementary Figure S6 RNA-seq analysis in proliferative and quiescent NPC cells.

**(A)** An experimental timeline for multiple schedules of paclitaxel/doxycycline treatment in cells (HONE1 TRE-SOX1 or CNE2 TRE-SOX1). The cells were treated with 1 µg/ml doxycycline (red block) to overexpress SOX1. The “Proliferation” group cells were treated with doxycycline for more than 14 days. The “Quiescence” group cells were pre-treated with doxycycline for 4 days and terminated on day 9. Meanwhile, cells were treated with 200 nM paclitaxel during the indicated timeline. Both groups of cells were collected and used for RNA-seq/WB analysis. **(B)** PCA of gene expression profiles in proliferative and quiescent NPC cells (HONE1 TRE-SOX1 or CNE2 TRE-SOX1). **(C)** Volcano diagram of the differentially expressed genes (DEGs) between proliferative and quiescent NPC cells (HONE1 TRE-SOX1 or CNE2 TRE-SOX1). SOX1 is highlighted. **(D)** KEGG pathway maps showing up- (red) or down-regulated (blue) genes in ribosome pathway. Each cell represents the log2 ratio gene expression data of the “Quiescence” group normalized to the “Proliferation” group in NPC cells (HONE1 TRE-SOX1 or CNE2 TRE-SOX1).
