# Supplementary figures and images for: SOX1 acts as a tumor hypnotist rendering nasopharyngeal carcinoma cells refractory to chemotherapy
Source: Cell Death Discov. 2023 Jun 27;9:194. doi: 10.1038/s41420-023-01479-x (PMC10300072; doi:10.1038/s41420-023-01479-x)

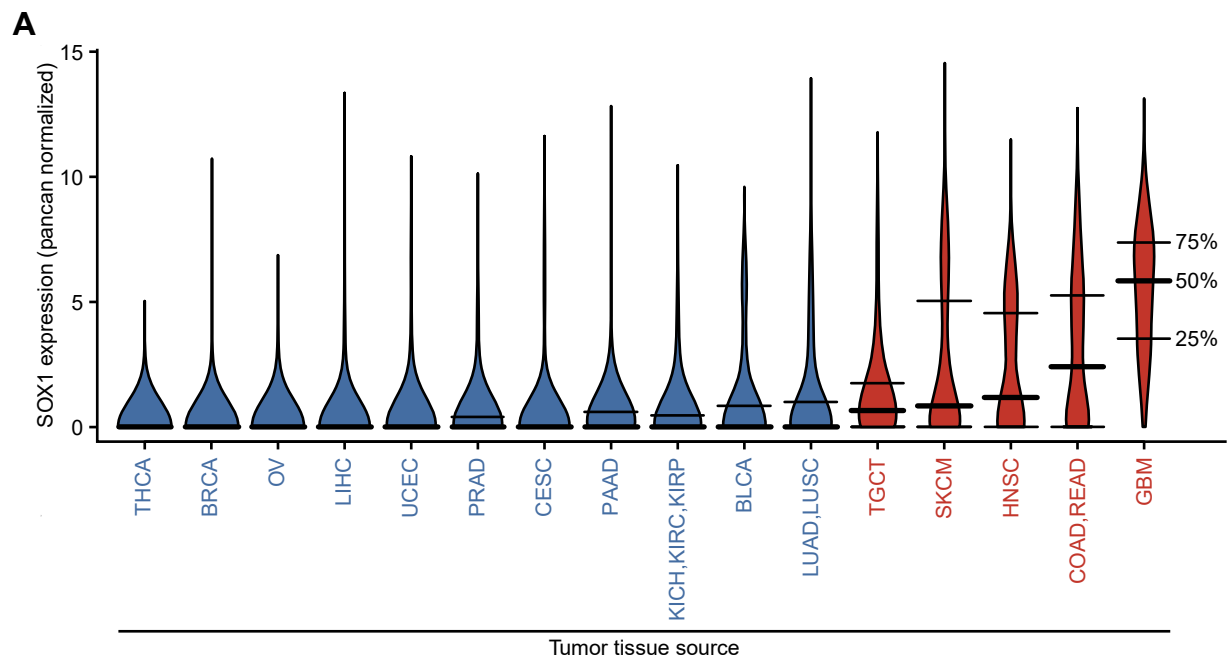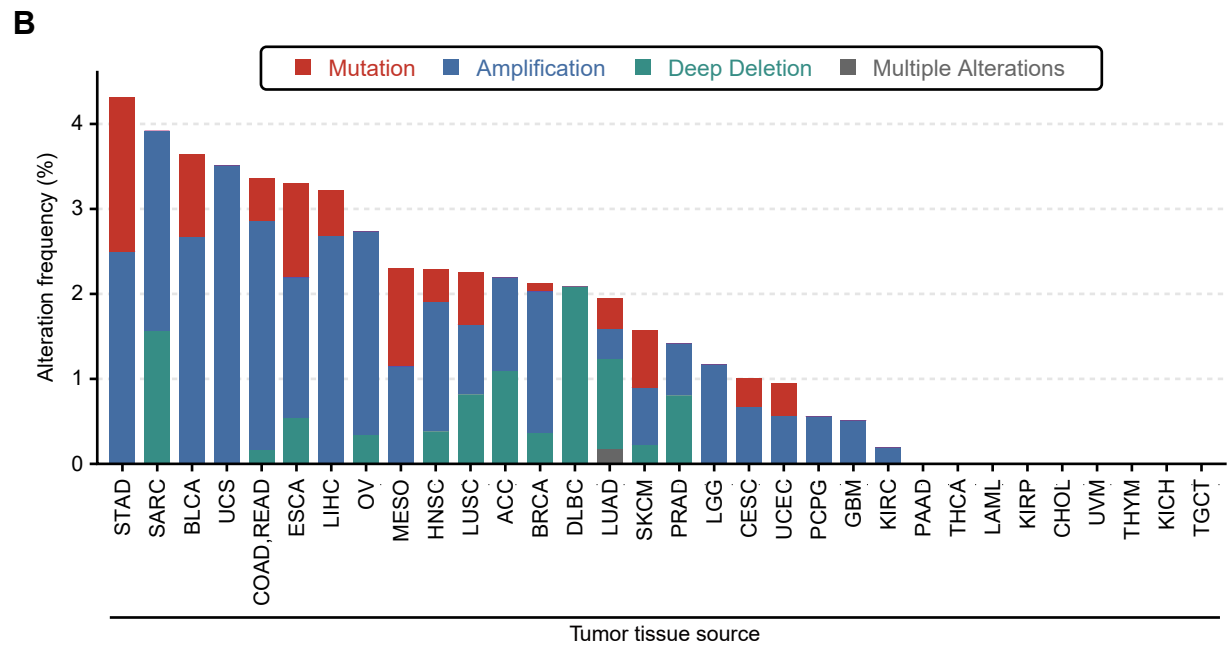

Supplement: Supplementary file 2 — Supplementary Figure S1 [file 41420_2023_1479_MOESM2_ESM.pdf]

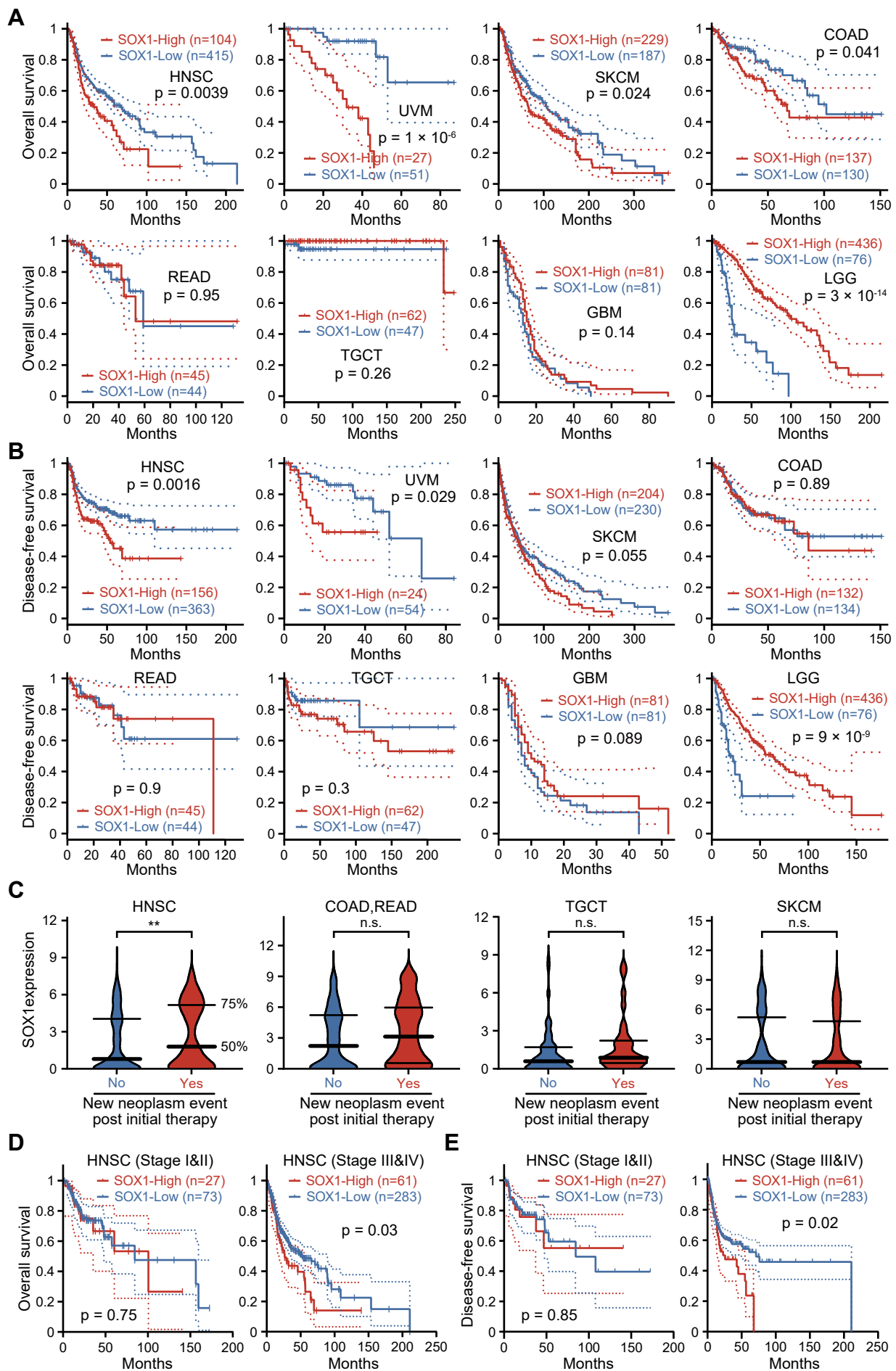

Supplement: Supplementary file 3 — Supplementary Figure S2 [file 41420_2023_1479_MOESM3_ESM.pdf]

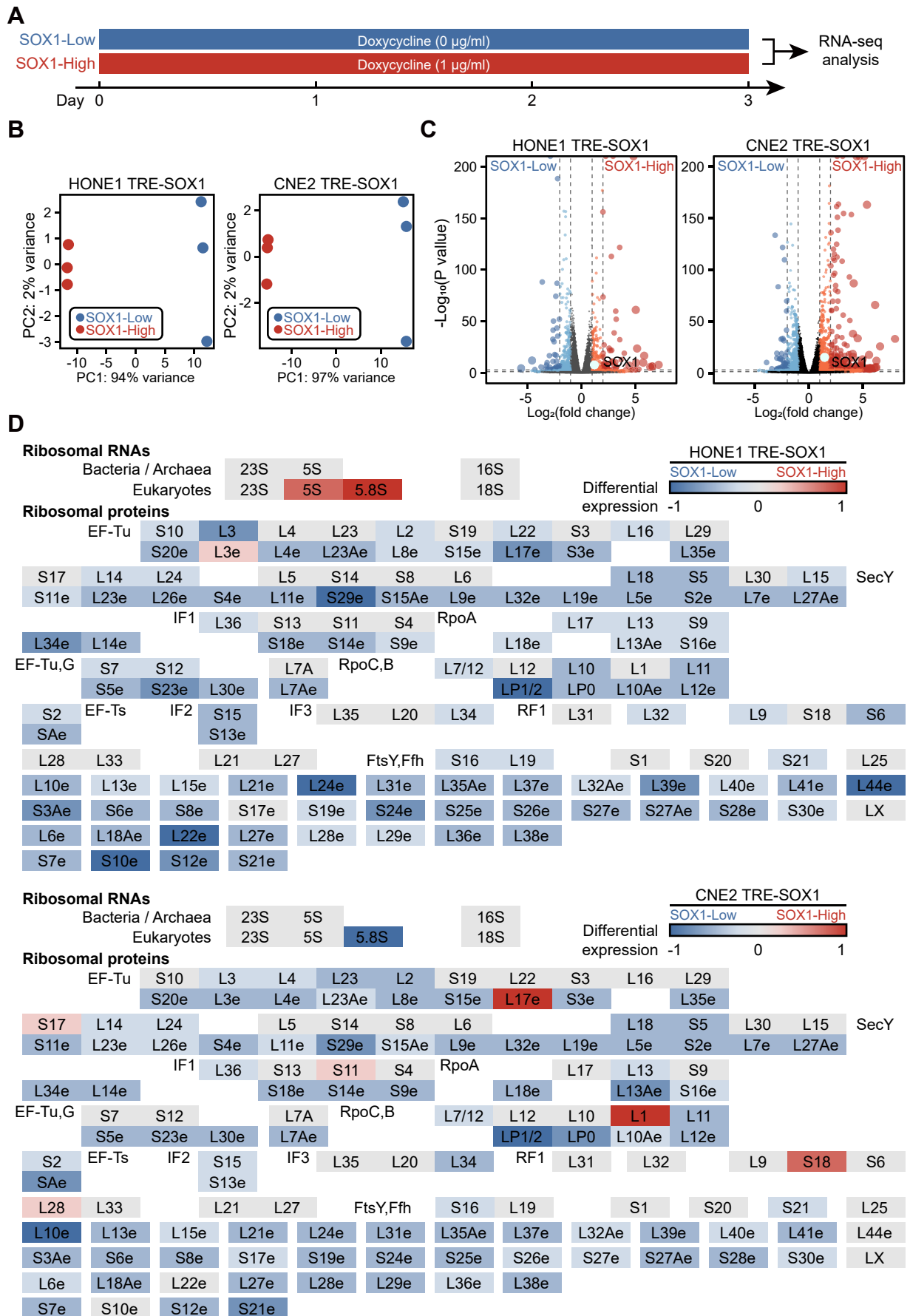

Supplement: Supplementary file 4 — Supplementary Figure S3 [file 41420_2023_1479_MOESM4_ESM.pdf]

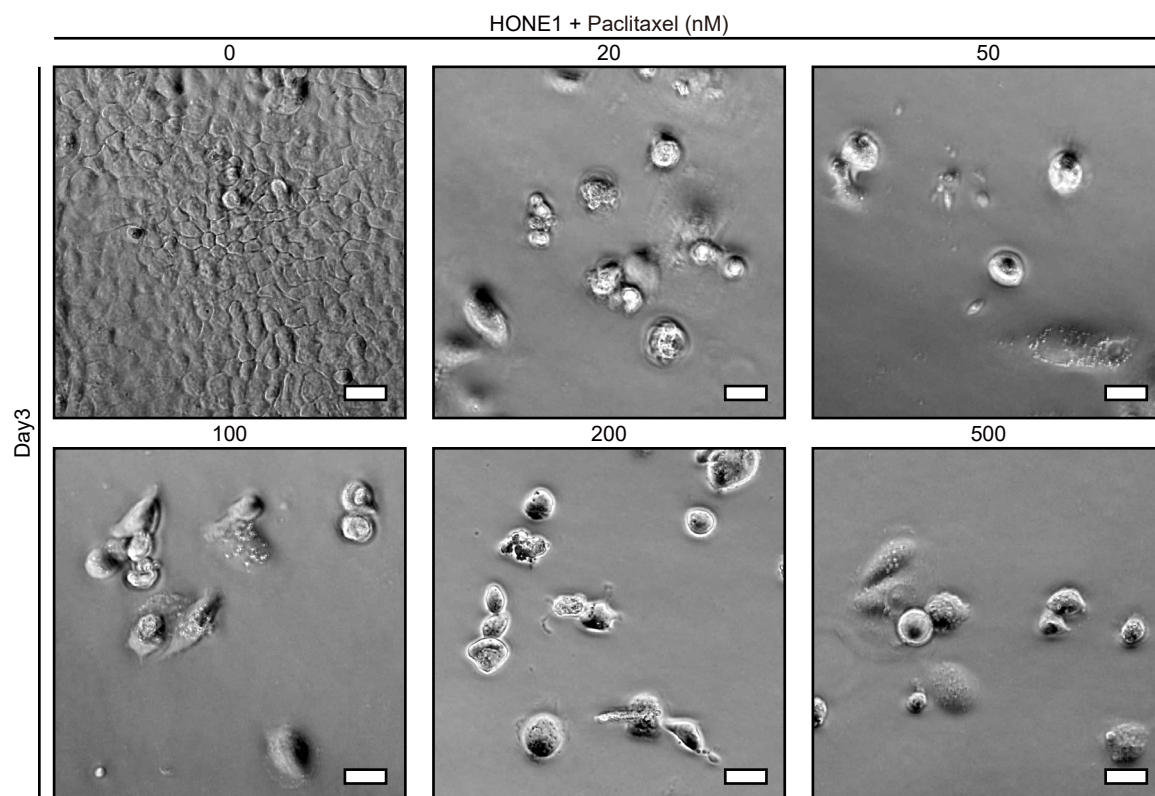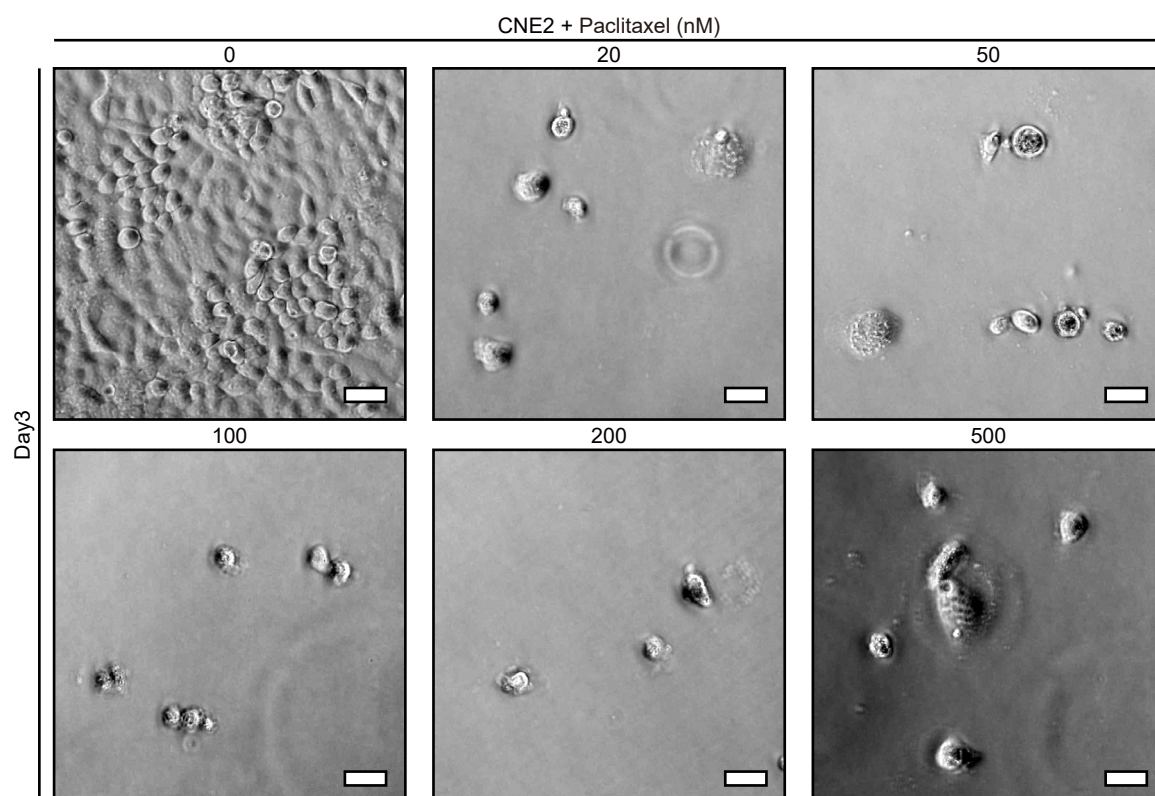

Supplement: Supplementary file 5 — Supplementary Figure S4 [file 41420_2023_1479_MOESM5_ESM.pdf]

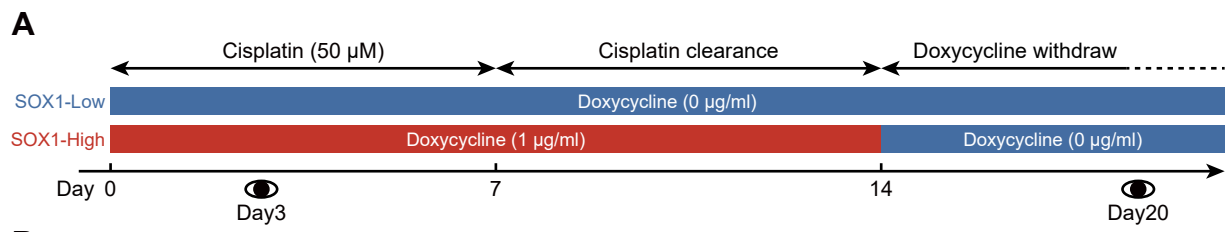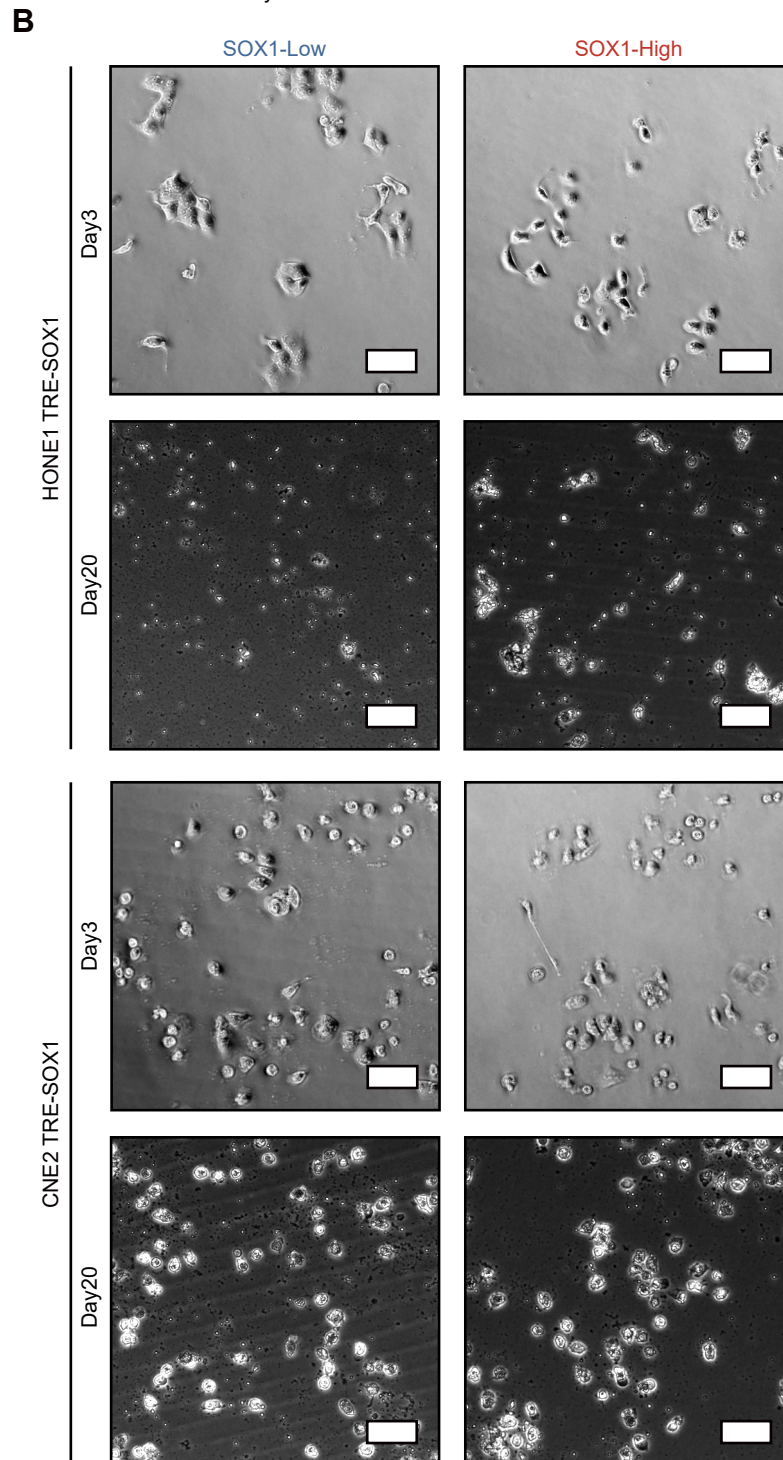

Supplement: Supplementary file 6 — Supplementary Figure S5 [file 41420_2023_1479_MOESM6_ESM.pdf]

**Fig. 1B**

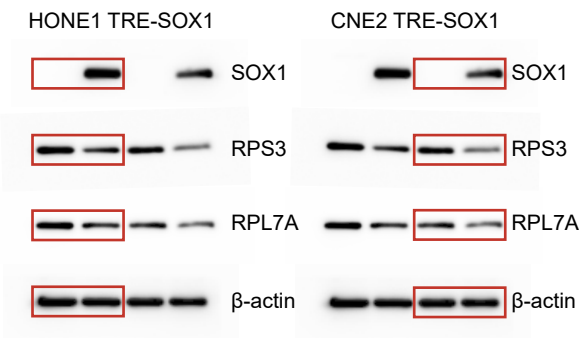

**Fig. 7A**

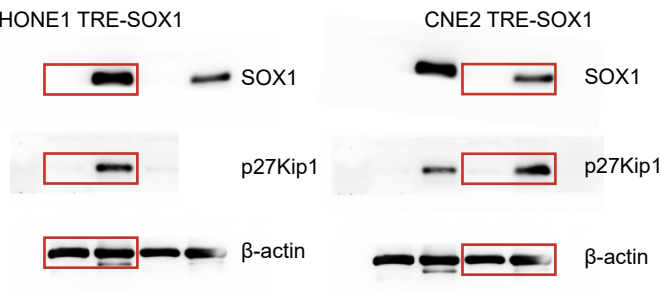

**Fig. 7C**

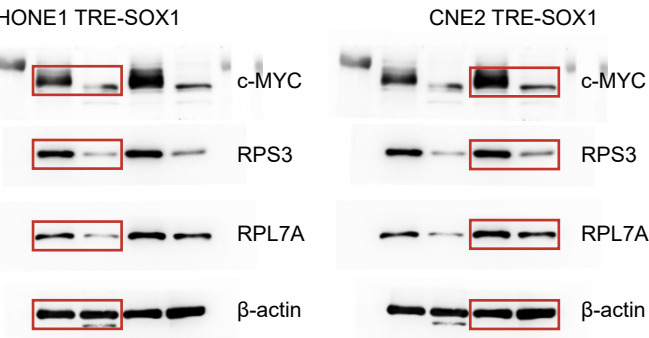

**Fig. 8B**

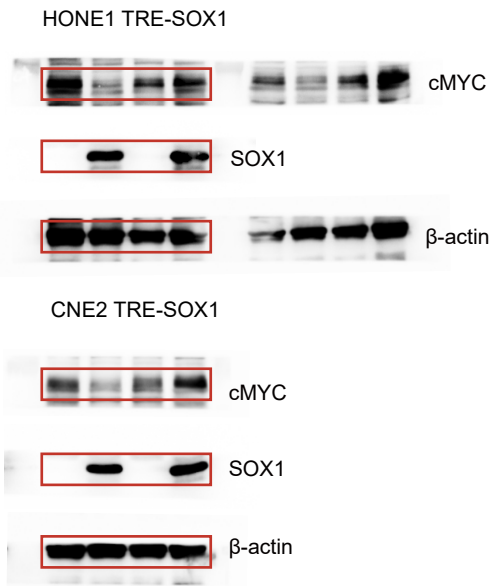

Supplement: Supplementary file 18 — Original western blots [file 41420_2023_1479_MOESM18_ESM.pdf]
