# Supplementary material for: SOX1 acts as a tumor hypnotist rendering nasopharyngeal carcinoma cells refractory to chemotherapy
Source: Cell Death Discov. 2023 Jun 27;9:194. doi: 10.1038/s41420-023-01479-x (PMC10300072; doi:10.1038/s41420-023-01479-x)
Supplement: Supplementary file 8 — Supplementary Table S1 [file 41420_2023_1479_MOESM8_ESM.docx]

**Supplementary Table S10. Primer sequences for plasmid construction**

| **Insertion name** | **Forward primer (5'-3')** | **Reverse primer (5'-3')** |
| --- | --- | --- |
| MYC promoter (0.5k) | CCTAACTGGCCGGTACCGCTAGCCGCGCGCCCATTAATACCCTTCTTTCCTC | AGATCTACGCGTGAGCTCCTCGAGCTCAGCCGTCCAGACCCTCGCAT |
| MYC promoter (1k) | CCTAACTGGCCGGTACCGCTAGCAGCCCGAGACTGTTGCAAACCGGCGCCACA | AGATCTACGCGTGAGCTCCTCGAGCTCAGCCGTCCAGACCCTCGCAT |
| MYC promoter (1.5k) | CCTAACTGGCCGGTACCGCTAGCATTTCAGGGAGCAAACAAATCATGTGTG | AGATCTACGCGTGAGCTCCTCGAGCTCAGCCGTCCAGACCCTCGCAT |
| MYC promoter (2k) | CTAACTGGCCGGTACCGCTAGCTGGAAACTTGTTTTAAGGAACCGC | AGATCTACGCGTGAGCTCCTCGAGCTCAGCCGTCCAGACCCTCGCAT |
| MYC promoter (MU1)  Fragment 1 | CTAACTGGCCGGTACCGCTAGCTGGAAACTTGTTTTAAGGAACCGC | TGTTGTTCCACGGCATGAAAAAAAAAAAACATTCTTCTCATCCTTGGTC |
| MYC promoter (MU1)  Fragment 2 | GACCAAGGATGAGAAGAATGTTTTTTTTTTTTCATGCCGTGGAACAACA | AGATCTACGCGTGAGCTCCTCGAGCTCAGCCGTCCAGACCCTCGCAT |
| MYC promoter (MU2)  Fragment 1 | CTAACTGGCCGGTACCGCTAGCTGGAAACTTGTTTTAAGGAACCGC | TATTCCCTCGGGATTTTTTATTTTTTGTTGTTCCACGGCATGAA |
| MYC promoter (MU2)  Fragment 2 | TTCATGCCGTGGAACAACAAAAAATAAAAAATCCCGAGGGAATA | AGATCTACGCGTGAGCTCCTCGAGCTCAGCCGTCCAGACCCTCGCAT |
