# Supplementary material for: SOX1 acts as a tumor hypnotist rendering nasopharyngeal carcinoma cells refractory to chemotherapy
Source: Cell Death Discov. 2023 Jun 27;9:194. doi: 10.1038/s41420-023-01479-x (PMC10300072; doi:10.1038/s41420-023-01479-x)
Supplement: Supplementary file 7 — Supplementary Figure S6 [file 41420_2023_1479_MOESM7_ESM.pdf]

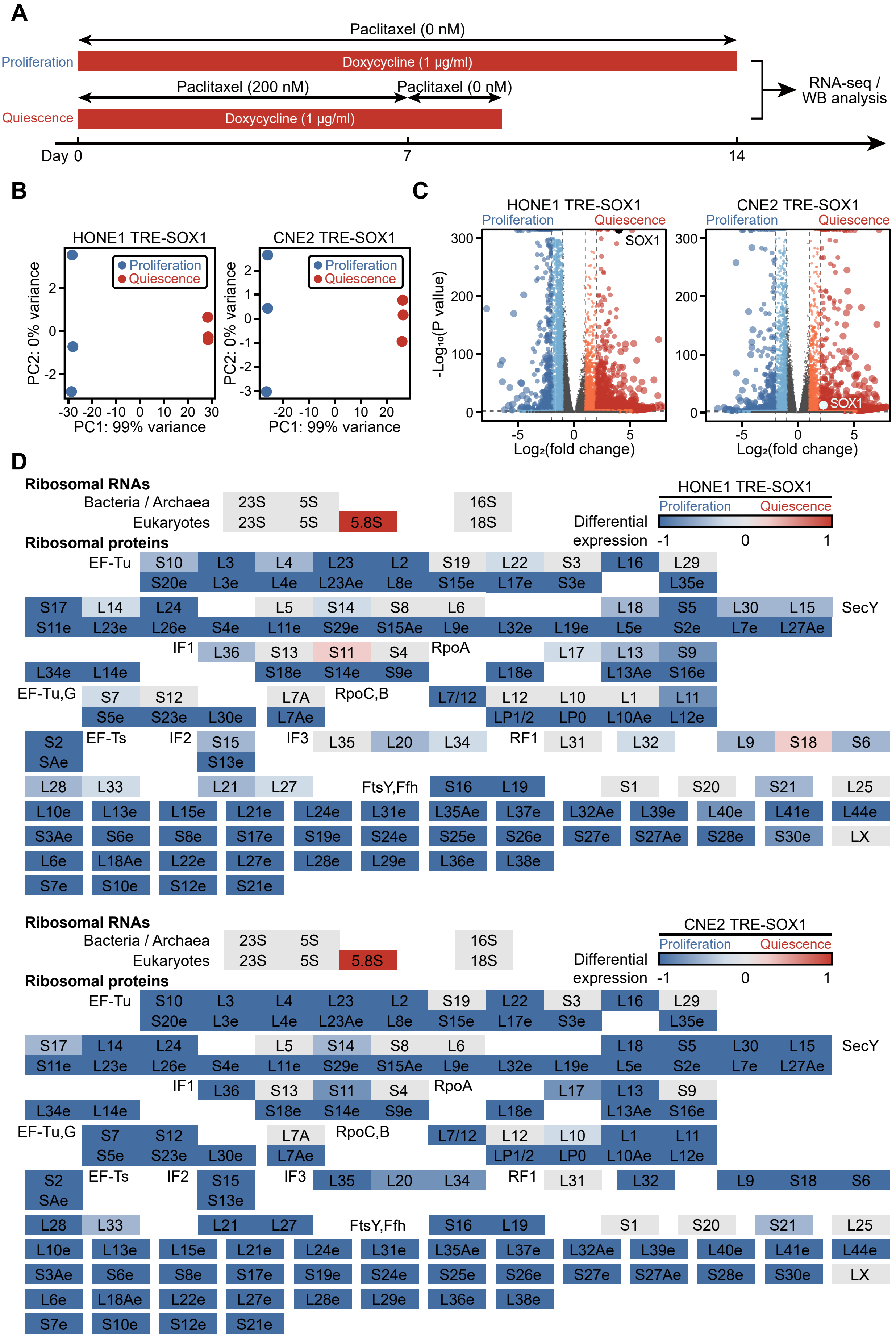
A
Paclitaxel (0 nM)
Proliferation
Doxycycline (1 μg/ml)
Paclitaxel (200 nM)
Paclitaxel (0 nM)
Quiescence
Doxycycline (1 μg/ml)
RNA-seq / WB analysis
Day 0
7
14
B
HONE1 TRE-SOX1
CNE2 TRE-SOX1
Proliferation
Quiescence
PC2: 0% variance
PC1: 99% variance
C
HONE1 TRE-SOX1
CNE2 TRE-SOX1
Proliferation
Quiescence
SOX1
-Log10(P value)
Log2(fold change)
D
Ribosomal RNAs
Bacteria / Archaea
Eukaryotes
23S 5S 16S
23S 5S 5.8S 18S
HONE1 TRE-SOX1
Proliferation
Quiescence
Differential expression
-1 0 1
Ribosomal proteins
EF-Tu S10 L3 L4 L23 L2 S19 L22 S3 L16 L29
S20e L3e L4e L23Ae L8e S15e L17e S3e L35e
S17 L14 L24 L5 S14 S8 L6 L18 S5 L30 L15 SecY
S11e L23e L26e S4e L11e S29e S15Ae L9e L32e L19e L5e S2e L7e L27Ae
IF1 L36 S13 S11 S4 RpoA L17 L13 S9
L34e L14e S18e S14e S9e L18e L13Ae S16e
EF-Tu,G S7 S12 L7A RpoC,B L7/12 L12 L10 L1 L11
S5e S23e L30e L7Ae LP1/2 LP0 L10Ae L12e
S2 EF-Ts IF2 S15 IF3 L35 L20 L34 RF1 L31 L32 L9 S18 S6
SAe S13e
L28 L33 L21 L27 FtsY,Ffh S16 L19 S1 S20 S21 L25
L10e L13e L15e L21e L24e L31e L35Ae L37e L32Ae L39e L40e L41e L44e
S3Ae S6e S8e S17e S19e S24e S25e S26e S27e S27Ae S28e S30e LX
L6e L18Ae L22e L27e L28e L29e L36e L38e
S7e S10e S12e S21e
Ribosomal RNAs
Bacteria / Archaea
Eukaryotes
23S 5S 16S
23S 5S 5.8S 18S
CNE2 TRE-SOX1
Proliferation
Quiescence
Differential expression
-1 0 1
Ribosomal proteins
EF-Tu S10 L3 L4 L23 L2 S19 L22 S3 L16 L29
S20e L3e L4e L23Ae L8e S15e L17e S3e L35e
S17 L14 L24 L5 S14 S8 L6 L18 S5 L30 L15 SecY
S11e L23e L26e S4e L11e S29e S15Ae L9e L32e L19e L5e S2e L7e L27Ae
IF1 L36 S13 S11 S4 RpoA L17 L13 S9
L34e L14e S18e S14e S9e L18e L13Ae S16e
EF-Tu,G S7 S12 L7A RpoC,B L7/12 L12 L10 L1 L11
S5e S23e L30e L7Ae LP1/2 LP0 L10Ae L12e
S2 EF-Ts IF2 S15 IF3 L35 L20 L34 RF1 L31 L32 L9 S18 S6
SAe S13e
L28 L33 L21 L27 FtsY,Ffh S16 L19 S1 S20 S21 L25
L10e L13e L15e L21e L24e L31e L35Ae L37e L32Ae L39e L40e L41e L44e
S3Ae S6e S8e S17e S19e S24e S25e S26e S27e S27Ae S28e S30e LX
L6e L18Ae L22e L27e L28e L29e L36e L38e
S7e S10e S12e S21e
